# The 2-oxoglutarate-dependent dioxygenase superfamily participates in tanshinone production in *Salvia miltiorrhiza*

**DOI:** 10.1093/jxb/erx113

**Published:** 2017-04-08

**Authors:** Zhichao Xu, Jingyuan Song

**Affiliations:** 1 Institute of Medicinal Plant Development, Chinese Academy of Medical Sciences & Peking Union Medical College, Key Laboratory of Bioactive Substances and Resources Utilization of Chinese Herbal Medicine, Ministry of Education, Beijing, China

**Keywords:** Hydroxylation, miltirone, *Salvia miltiorrhiza*, danshen, tanshinone biosynthesis, 2-oxoglutarate-dependent dioxygenase, 2OGD5 RNAi.

## Abstract

Highly oxidized tanshinones are pharmacological ingredients extracted from the medicinal model plant *Salvia miltiorrhiza* and are mainly used to treat cardiovascular diseases. Previous studies have confirmed that cytochrome P450 mono-oxygenases (CYP450s) have a key function in the biosynthesis of tanshinones; however, no solid evidence links oxidation to the 2-oxoglutarate-dependent dioxygenase (2OGD) superfamily. Here, we identified 132 members of the DOXB and DOXC subfamilies of 2OGD by scanning the 2OG-FeII Oxy domain using a genome-wide strategy in *S. miltiorrhiza*. The DOXC class was phylogenetically divided into twelve clades. Combining phylogenetic relationships, differential expression and co-expression from various organs and tissues revealed that two 2OGDs were directly related to flavonoid metabolism, and that 13 2OGDs from different clades were predicted to be involved in tanshinone biosynthesis. Based on this insight into tanshinone production, we experimentally detected significant decreases in miltirone, cryptotanshinone, and tanshinone IIA (0.16-, 0.56-, and 0.56-fold, respectively) in *2OGD5* RNAi transgenic lines relative to the control lines using a metabonomics analysis. 2OGD5 was found to play a crucial role in the downstream biosynthesis of tanshinones following the hydroxylation of CYPs. Our results highlight the evolution and diversification of 2OGD superfamily members and suggest that they contribute to the complexity of tanshinone metabolites.

## Highlight

We identified and phylogenetically classified 132 2-oxoglutarate-dependent dioxygenase (2OGD) superfamily members in *Salvia miltiorrhiza*, and showed that 2OGD5 is involved in tanshinone biosynthesis.

## Introduction

*Salvia miltiorrhiza* (danshen), which is one of the most widely used herbs in traditional Chinese medicine, has been described as an ideal model medicinal plant for the study of biosynthesis and regulation of secondary metabolism, especially the molecular mechanism of diterpene biosynthesis (Song *et al.*, 2013; Xu *et al.*, 2016*a*, 2016*b*). Tanshinones are highly oxidized natural products from the roots/rhizome of *S. miltiorrhiza*, and their biosynthesis is based on the general diterpenoid precursor (E,E,E)-geranylgeranyl diphosphate (GGPP), which is produced via the mevalonate (MVA) and 2-C-methyl-D-erythritol 4-phosphate (MEP) pathways. The diterpenoid synthases copalyl diphosphate synthetase (CPS) and kaurene synthase (KS) catalyse GGPP into miltiradiene, the tanshinone backbone (Zhou *et al.*, 2012; Cui *et al.*, 2015). As has been shown in previous studies, cytochrome P450 mono-oxygenases (CYP450s) play the most important role in the downstream modification of tanshinone biosynthesis. CYP76AH1, CYP76AH3, and CYP76AK1 have been shown to be involved in the catalytic biosynthesis of ferruginol, dihydroxy ferruginol, and dihydroxy sugiol, respectively, which are intermediate products involved in tanshinone biosynthesis (Guo *et al.*, 2013, 2016). Although significant progress in analysing the tanshinone pathway has been made, many oxidation steps involved in the biosynthesis of downstream metabolites remain unknown. In addition, it has not been determined whether other oxidase families play an important role in tanshinone biosynthesis. In plants, at least 136 diterpenoid gibberellin (GA) structures have been reported, and the 2-oxoglutarate-dependent dioxygenase (2OGD) superfamily catalyses the transformations of many GA molecules (Lange *et al.*, 1994; Han and Zhu, 2011). These findings suggest that the 2OGD superfamily might participate in tanshinone production in *S. miltiorrhiza* (Zi *et al.*, 2014; Xu *et al.*, 2015).

In plant secondary metabolism, oxygenases play particularly important roles in oxygenation or hydroxylation reactions. Understanding the detailed biosynthesis pathway is necessary to construct synthesis modules to produce specified compounds *in vitro*. The vast majority of the downstream metabolic pathways remain untapped. The CYP450 and 2OGD superfamilies, which are two of the largest gene families involved in plant metabolism, mainly participate in the oxydic processes of natural products, especially terpenoid compounds. The CYP450 superfamily, which consists of heme-thiolate membrane proteins, are excellent reporters for plant metabolism evolution and have a key function in chemodiversity (Mizutani and Ohta, 2010; Boutanaev *et al.*, 2015). 2OGDs can typically catalyse the oxidation of an organic substrate using a dioxygen molecule, primarily by using a ferrous iron [Fe(II)] as the active site co-factor and 2OG as a co-substrate that is decarboxylated to succinate and CO_2_ (R + 2OG + O_2_ → R-OH + succinate + CO_2_) (Kawai *et al.*, 2014). This catalytic reaction of 2OGDs is related to multiple biological processes, including proline hydroxylation, the oxidative demethylation of nucleic acids and histones, and the biosynthesis of many metabolites (e.g. GAs and flavonoids). The plant 2OGD superfamily can be classified into three subfamilies according to phylogenetic function: DOXA, DOXB, and DOXC. In Arabidopsis, the DOXA class contains 14 2OGD members, which are described as DNA repair proteins and function in N-methyl group hydroxylation (Falnes *et al.*, 2002). The DOXB class includes 14 prolyl 4-hydroxylases (P4Hs), which are involved in the modification of proline hydroxylation of cell wall proteins (Keskiaho *et al.*, 2007). The largest class, DOXC, is composed of 100 2OGD members involved in the biosynthesis of secondary metabolites, such as GA 2-oxidases (GA2oxs), GA 3β-hydroxylases (GA3oxs), GA 20-oxidases (GA20oxs), flavanone 3-hydroxylases (F3Hs), anthocyanidin synthases (ANSs), 1-aminocyclopropane carboxylic acid oxidases (ACOs), dioxygenases for auxin oxidation (DAOs), and glucosinolate biosynthesis (AOPs).

The production and accumulation of tanshinone pigments are localized in the root periderm, which provides an effective system for studying candidate modified enzymes during tanshinone biosynthesis (Xu *et al.*, 2015). Many candidate CYP450s have been proposed to potentially catalyse oxygenation or hydroxylation reactions in tanshinone biosynthesis based on differential gene expression in various organs or tissues (Xu *et al.*, 2015, Xu *et al.*, 2016*b*). However, the 2OGD superfamily in *S. miltiorrhiza* has not been systematically studied using a genome-wide strategy (Chen *et al.*, 2015), and the following questions can be asked. Do candidate 2OGDs have a real, specific function in determining the oxidative activity of tanshinones? In *S. miltiorrhiza*, which contains highly oxidative products, do differences in the evolutionary status of the 2OGD oxidase superfamily exist compared with those of, for example, *Arabidopsis*, *Oryza*, and *Selaginella*?

In our study, the gene members, structures, phylogenetic tree, and homologous functions of the 2OGD superfamily in *S. miltiorrhiza* were systematically analysed. Candidate 2OGD genes involved in tanshinone biosynthesis were further identified according to gene-function enrichment analysis and gene expression profiles from different organs and tissues. In addition, transgenic RNAi hairy root systems of candidate 2OGD genes were constructed to verify their functional role in tanshinone biosynthesis. These results revealed the functional role of 2OGDs in the tanshinone biosynthesis pathway and provide a new foundation for investigating the biosynthesis of highly oxidized natural products.

## Materials and methods

### *Genome-wide survey of 2OGD genes in* S. miltiorrhiza

The Arabidopsis 2OGD protein sequences (AtGA20ox, AtGA3ox, AtGA2ox, and ACO) were downloaded from the National Center for Biotechnology Information (NCBI) database (http://www.ncbi.nlm.nih.gov/protein/). BLASTP searches were performed to identify the corresponding 2OGD gene members in *S. miltiorrhiza* using a cut-off *e*-value of 1.0 × 10^–10^ (Xu *et al.*, 2016*a*). The 2OG-FeII_Oxy motif (PF03171) was applied to identify 2OGD gene family members based on hidden Markov model (HMM) profiles. The Compute pI/Mw tool on the ExPASy server (http://web.expasy.org/compute_pi/) was employed to predict the theoretical isoelectric point (pI) and molecular weight (Mw) of each 2OGD protein. The Gene Structure Display Server (GSDS 2.0, http://gsds.cbi.pku.edu.cn/index.php) was used to analyse the gene structures of 2OGDs using the input coding sequences (CDSs) and corresponding genomic sequences. Conserved motifs in 2OGD proteins were identified using MEME (Suite version 4.9.1, http://meme-suite.org/tools/meme) according to the following criteria: maximum number of motifs, 10; optimum width, 8–50 amino acids. The subCELlular LOcalization predictor (CELLO v.2.5, http://cello.life.nctu.edu.tw/) was used to predict the subcellular localization of 2OGD proteins.

### Phylogenetic tree construction and homology modeling

All annotated 2OGD protein sequences from *Arabidopsis thaliana*, *Oryza sativa*, and *S. miltiorrhiza* were pooled into MEGA6 (http://www.megasoftware.net/), and multiple sequence alignments were performed. Then, neighbor-joining trees were constructed using the bootstrap method with 1000 replications and the pairwise deletion of gaps/missing data. Following the classification of At2OGDs and Os2OGDs, systematic classes of Sm2OGDs were identified using the phylogenetic tree. CLANS was used to cluster and connect the 2OGD protein sequences with *P*-values <10^–4^ (http://www.eb.tuebingen.mpg.de/research/departments/protein-evolution/software/clans.html) (Frickey and Lupas, 2004). The homology model of 2OGD5 was constructed using Discovery Studio v2.5 (http://accelrys.com/products/collaborative-science/biovia-discovery-studio/), and the homology model and binding sites were visualized using PyMOL (http://pymol.org).

### Plant resources and transcriptomic analysis

*Salvia miltiorrhiza* (line 99-3) was cultivated at the Institute of Medicinal Plant Development (IMPLAD), Chinese Academy of Medical Sciences (CAMS), in an open experimental field. All of the collected tissues originated from an asexual line of *S. miltiorrhiza* 99-3.

The genome of *S. miltiorrhiza* was assembled and annotated in our lab (SRP051524) (Xu *et al.*, 2016*a*). The RNA-seq reads from different organs root, stem, and flower), different root tissues (periderm, phloem, and xylem), and leaves with or without 12-h MeJA (methyl jasmonate) treatment were sequenced in our previous studies (SRR1640458, SRP051564, and SRP028388) (Luo *et al.*, 2014; Xu *et al.*, 2015, 2016*a*; Zhang *et al.*, 2015; Ji *et al.*, 2016). Subsequently, all the raw data were combined for differential expression analysis using Tophat 2.0.12 and Cufflinks 2.2.1 (Trapnell *et al.*, 2012). A heat map was constructed using the R statistical software (https://www.r-project.org). Gene ontology (GO) mapping and annotation were performed using Blast2GO with a cut-off *e*-value of 1.0 × 10^–10^.

### *RNAi transgenic hairy roots of* S. miltiorrhiza

The 186-bp gene fragments of 2OGD5 (loci: 304–489 bp) were specifically selected from the genomic data and cloned into the PMD18-T vector with attB primers (see Supplementary Table S1 at *JXB* online) from Gateway Technology (Invitrogene, USA). The attB PCR products were subcloned into the donor vector pDONR221 using the BP clonase II enzyme. Then, LR recombination reactions between pDONR221-2OGD5 and the destination vector pK7GWIWG2D(II) were conducted to generate a binary vector with green fluorescent protein (GFP) expression sequences. The pK7GWIWG2D(II)-2OGD5 RNAi vector was transformed into *Rhizobium rhizogenes* ACCC10060, which was used to infect the *S. miltiorrhiza* leaves and induce the formation of hairy roots. The empty ACCC10060 and ACCC10060 with pK7GWIWG2D(II) vectors were transfected into leaves as negative controls (‘CK’ and ‘PK’, respectively). The callus and hairy roots appeared at approximately 7–10 d after infection. Then, 2-cm long hairy roots were cut off and stimulated with 0.1 mg l^–1^ indole-3-acetic acid (IAA) to produce lateral roots. The transgenic hairy roots were mass cultured for qPCR and metabolite analyses in 6, 7-V liquid medium at 25 °C for up to 2 months in the dark.

### Metabolite analysis

For ultra-performance liquid chromatography (UPLC) analysis, the fresh hairy roots were dried at 45 °C and ground into a powder by ball milling. In total, 1 g of powder was extracted in 5 ml of methanol. Then, the mixture was sonicated for 15 min and centrifuged at 2500 *g* for 10 min, and the supernatant was filtered using a 0.22-μm polytetrafluoroethylene syringe. Subsequently, 16 μg each of tanshinone I, tanshinone IIA, dihydrotanshinone I, cryptotanshinone, ferrugiol, and miltirone were dissolved in 1 ml of methanol to produce reference standards. Chromatographic separations were performed using the Waters ACQUITY UPLC BCH C18 column with a mobile phase of 75% methanol:25% H_2_O in a Waters UPLC system (Waters, USA).

For UPLC-MS/MS analysis, another Shim-pack UFLC SHIMADZU CBM20A UPLC system was used with a Waters ACQUITY UPLC HSS T3 C18 (1.8 μm, 2.1 × 100 mm) column. The mobile phase was acetonitrile (0.04% acetic acid, A) and H_2_O (0.04% acetic acid, B). The UPLC program had a linear gradient from 5% to 95% A (0–12 min) and then 95% A (12–15 min). The flow rate was 0.4 ml min^–1^, the column temperature was set to 40 °C, and the injected volume of each sample was 4 μl. The MS/MS analysis was performed using a Biosystems 4500 QTRAP. The parameters were set as follows: the electrospray ionization (ESI) temperature was 550 °C, the voltage was 5500 V, the curtain gas (CUR) was 25 psi (172 kPa), and the collision-activated dissociation (CAD) was set to high. The raw data (wiff format) were processed with Analyst 1.6.1 (AB SCIEX).

### Gene expression analysis by qRT-PCR

Total RNA was isolated from three biological replicates of each sample using the RNeasy Plus Mini kit (Qiagen, Germany). Reverse transcription was performed using PrimeScript™ Reverse Transcriptase (TaKaRa, Japan). The qRT-PCR primers were designed using Primer Premier 6 (see Supplementary Table S1), and their specificity was verified by PCR. qRT-PCR analysis was conducted in triplicate using SYBR® Premix Ex Taq™ II (TaKaRa, Japan), with SmActin as a reference gene using a LightCycler 480 real-time PCR system (Roche, Switzerland). Ct values were calculated to analyse the relative expression levels using the 2^–ΔΔCt^ method. To detect differences in the expression of candidate genes, a one-way analysis of variance (ANOVA) was performed using IBM SPSS 20 software (IBM Corporation, USA).

## Results

### *Genome identification of 2OGDs in* S. miltiorrhiza

A total of 132 putative 2OGD sequences were annotated by scanning the 2OG-FeII_Oxy domain (PF03171) against the whole amino acid sequences of *S. miltiorrhiza*. The gene number of 2OGD superfamily genes was similar to the number known in seed plants such as *Arabidopsis thaliana* (130), *Oryza sativa* (114), and *Picea abies* (142). The predicted 2OGD proteins varied from 120 amino acids (SMil_00019454) to 504 amino acids (SMil_00021498), with corresponding molecular weights from 13.62 kDa to 55.76 kDa, and the theoretical isoelectric points ranged from 4.63 (SMil_00021251) to 9.28 (SMil_00019454). Pairwise analysis of 2OGD homology indicated that the overall homology broadly ranged from 29.07% (between SMil_00005410 and SMil_00013745) to 99.64% (between SMil_00008540 and SMil_00008541). Most of the gene locations determined using TargetP prediction were unknown (see Supplementary Table S2). Gene structure analysis of the 2OGD genes revealed that the number of exons ranged from 1 to 15; however, SMil_00018082 and SMil_00029434 were intronless (Supplementary Fig. S2). The gene width localized in the genome range from 591 bp to 7.76 kb, and the longest 2OGD (SMil_00011070) included 15 exons.

### Phylogenetic analysis and diversity of 2OGDs

**Fig. 1.**
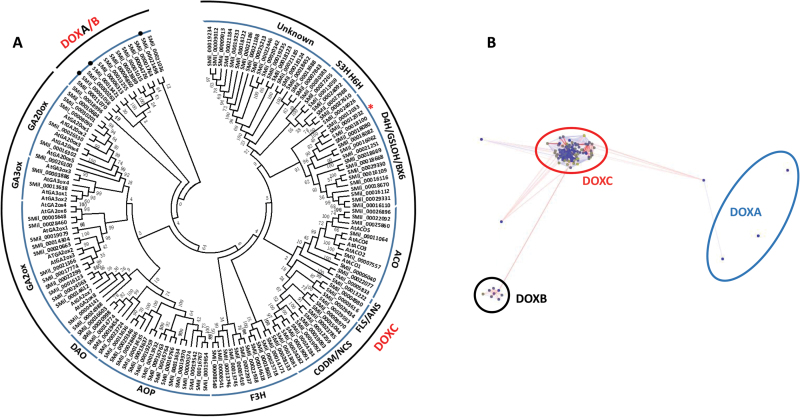
(A) Analysis of the phylogenetic relationships of *2OGD* gene members in *S. miltiorrhiza*. A total of 132 2OGD proteins from *S. miltiorrhiza* were used to construct a neighbor-joining tree. Bootstrap values are presented for all branches. The 132 2OGDs were clustered into two classes, and then the DOXC class was divided into 11 clades. These clades were named according to their known functions in other species. The asterisk (*) indicates *2OGD5*, on which functional analysis was performed. (B) The CLANS analysis clustered the 2OGDs into three classes (DOXA, DOXB, and DOXC). Three 2OGD members were classified into the DOXA class, which differed from the phylogenetic tree. Connected dots indicate significant similarity (*P*<10^–4^) based on the BLASTP search. The other three dots outside of the DOXA–C clusters were clustered in the unclassified group.

All 2OGD sequences were divided into three functionally distinct classes, which are named DOXA, DOXB, and DOXC in other organisms. Only two classes were identified in *S. miltiorrhiza* from the phylogenetic tree (Fig. 1A). BLASTP and CLANS were used to further confirm the classification of 2OGDs in *S. miltiorrhiza*; however, the results showed that three 2OGDs, namely SMil_00001058, SMil_00013421, and SMil_00021498, were classified into the DOXA class (Fig. 1B, Supplementary Table S3). There were considerably fewer DOXA gene members in *S. militiorrhiza* than in other plants, such as *A. thaliana* and *O. sativa.* The numbers of 2OGD genes in the DOXA, DOXB, and DOXC classes were extremely variable in *S. miltiorrhiza*, and the DOXC proteins in the 2OGD superfamily were dominant. Eight 2OGD proteins clustered in the DOXB class, and the other 118 2OGDs were categorized as DOXC. Additionally, three 2OGDs (SMil_00000263, SMil_00006313, and SMil_00011070) were identified as an unclassified group. All the members of DOXB in *S. miltiorrhiza* were annotated as P4H proteins and were predicted to hydroxylate prolines to form hydroxyproline-rich glycoproteins during the synthesis of the plant cell wall. The 2OGD proteins from the DOXC class were determined to be related to the biosynthesis of specialized metabolites, such as GAs and flavonoids in seed plants. According to their functional diversity and phylogenetic distribution, 118 2OGDs were mainly clustered into 11 protein families in the DOXC class, including GA20ox (6), GA3ox (2), GA2ox (13), DAO (5), AOP (16), F3H/FLS/ANS (15), CODM/NCS (11), ACO (8), D4H/GSLOH/BX6 (17), H6H (3), S3H (2), and Unknown (19). Most of the DOXC classes contained two domains: the DIOX_N domain and the 2OG-FeII_Oxy domain. The gene number in the DOXC clade was more extensive in *S. miltiorrhiza* than in *A. thaliana* (96), *O. sativa* (89), *P. abies* (61), *Selaginella moellendorffii* (31), *Physcomitrella patens* (13), and *Chlamydomonas reinhardtii* (2) (Kawai *et al.*, 2014). ACO proteins catalyse the transformation of 1-aminocyclopropane carboxylic acid to ethylene and were specifically found in angiosperms and gymnosperms. The phylogenetic tree of *S. miltiorrhiza* included five ACO genes clustered with the ACOs of *A. thaliana*, suggesting that ethylene biosynthesis is conserved in higher plants. Other phytohormone metabolic pathways in *S. miltiorrhiza*, such as those for auxin and GA biosynthesis, also rely on the catalysed function of 2OGDs, indicating that *de novo* evolution from lower to higher plants occurred. The largest DOXC clade in *S. miltiorrhiza* was the D4H/GSLOH/BX6 clade, which is also the major clade in *A. thaliana*. This clade has been suggested to be involved in aliphatic glucosinolate biosynthesis (GSLOH), benzoxazinoid biosynthesis (BX6), and monoterpenoid indole alkaloid biosynthesis (D4H). This DOXC clade contained diverse 2OGD functions involved in the production of lineage-specific secondary metabolites to defend against various stresses.

In total, ten conserved motifs in 2OGDs were characterized (motifs 1–10) using MEME software to explore their structures and functional diversity. Motifs 2, 4, 6, 1, and 9 constitute the conserved 2OGD-FeII Oxy domain, and all the 2OGDs contained at least one of these five motifs. The motif locations revealed that most of the 2OGD-FeII_Oxy motifs are near the C-terminal in 2OGDs (Supplementary Figs S3 and S4). Among them, eight 2OGDs from the DOXB class (SMil_00004726, SMil_00021498, SMil_00001764, SMil_ 00006313, SMil_00000263, SMil_00018996, SMil_00001015, and SMil_00013421) contained only 1–3 motifs, and their *e*-values exceeded 10^–10^, suggesting that these DOXB 2OGDs underwent an evolutionary process distinct from that of other DOXC 2OGDs.

To investigate the biological processes involving 2OGDs, GO mapping and annotation were performed using Blast2GO. The functional categorization of 2OGDs as annotated by GO analysis, including their biological processes, molecular functions, and cellular components, is presented in Supplementary Fig. S5. Regarding biological processes, four categories met the criterion of NodeScore >1.0: single-organism cellular processes (12 genes), single-organism metabolic processes (129 genes), cellular metabolic processes (14 genes), and organic substance metabolic processes (20 genes). Among the metabolic-process genes, all 129 were described as being involved in secondary metabolic processes. Based on the molecular function analysis, 130 2OGDs were classified according to their oxidoreductase activity, and the NodeScores were as high as 210. Based on the cellular component analysis, six 2OGDs from the P4H family were functionally localized in parts of cells, which is in agreement with their known roles in the synthesis of the plant cell wall.

### Expression patterns of 2OGD genes in different organs and tissues

**Fig. 2.**
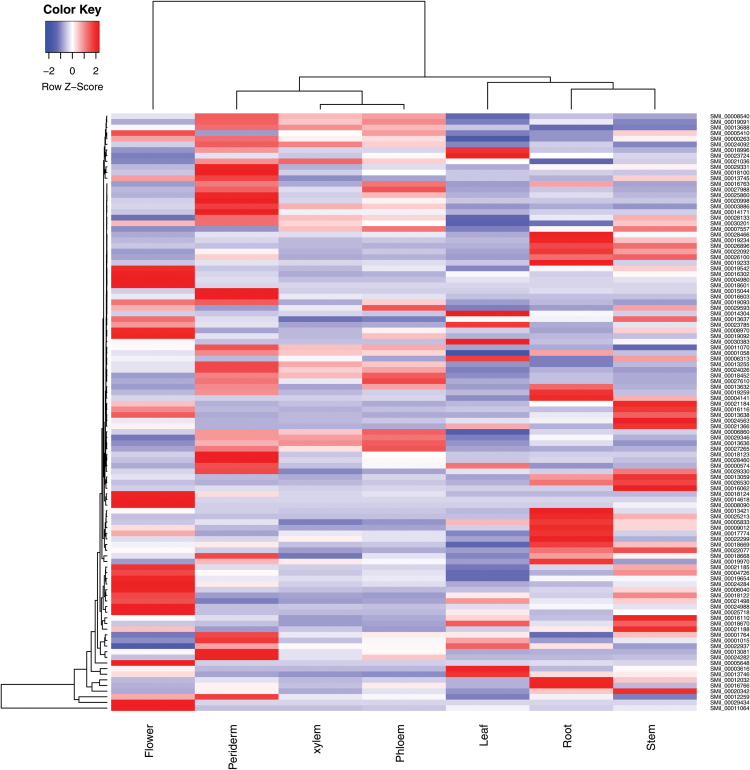
Heat map depicting *2OGD* gene expression patterns in different organs and root tissues. The colors represent decreasing log_10_(RPKM) values as indicated in the key.

To better investigate the oxidative activities of 2OGDs in *S. miltiorrhiza*, the differential expression of 132 2OGD genes in different organs (leaf, root, stem, and flower), root tissues (periderm, phloem, and xylem), and after MeJA treatments (1 h and 12 h) was determined by analysing the RNA-seq data (see Supplementary Table S4). The values for reads per kilobase of transcript per million mapped reads (RPKM) of 26 2OGD genes in all samples were less than 1, and these genes were considered to be silencing genes or pseudogenes. Among them, 25 2OGD silencing genes were derived from the DOXC class. The gene expression levels of the other 106 2OGD genes are shown in Fig. 2. The tissue-specific expression analysis indicated that SMil_00014618 and SMil_00029434 exhibited unique expressions in flower tissues. These two genes were clustered into the F3H and FLS/ANS clades, respectively, but both were involved in flavonoid metabolism. Here, SMil_00014618 was identified as the enzyme that catalyses flavanones to dihydroflavonols and, subsequently, SMil_00029434 hydroxylates the dihydroflavonols to produce anthocyanidin. Two 2OGDs (SMil_00016766 and SMil_00019970) presented root-specific expression, and no 2OGD genes were specifically expressed in the leaf and stem. The expression levels of 10 2OGDs genes increased significantly after MeJA treatment (12 h).

### Candidate 2OGDs related to tanshinone biosynthesis

**Fig. 3.**
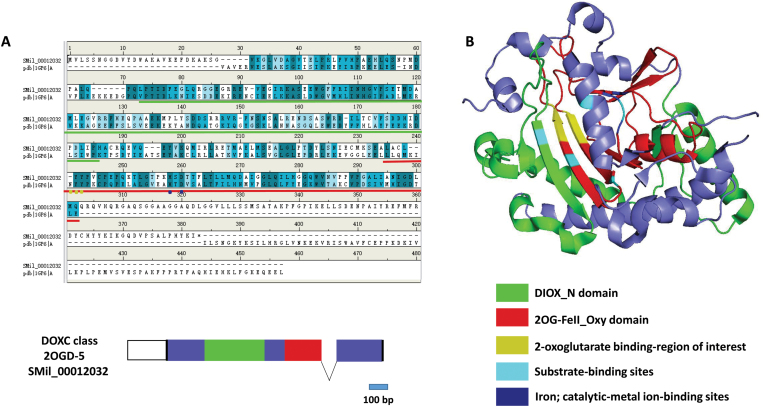
The gene structure and homology modeling of *2OGD5* from *S. miltiorrhiza*. (A) *2OGD5* was aligned to the anthocyanidin synthase sequence of *A. thaliana* (AtLDOX). (B) The predicted homology modeling was derived from the PDB database (PDBID:1GP6). The different colors represent the different domains and binding sites, as indicated in the key.

In *S. miltiorrhiza*, both tanshinone accumulation and biosynthesis occur in the periderm tissue. Therefore, candidate 2OGDs related to tanshinone biosynthesis were selected according to their gene co-expression in different organs, root tissues, and after MeJA treatments. Thirteen 2OGDs were retrieved based on the following criteria: periderm/phloem RPKM >1.5, phloem/xylem RPKM >1.5, and periderm RPKM >10 (see Supplementary Table S5). Interestingly, all the candidate 2OGDs belonged to the DOXC class, and six 2OGDs of the 13 candidate genes were from the D4H/GSLOH/BX6 clade. However, tanshinones are mainly produced in the root and rhizome of *S. miltiorrhiza*, and only one 2OGD gene showed significantly high expression in roots (*2OGD5*, SMil_00012032, Supplementary Table S5). Moreover, the expression of *2OGD5* was up-regulated by the induction of MeJA, which was positively correlated with tanshinone accumulation after MeJA treatment. The gene structure of *2OGD5* included two exons, and both the DIOX_N domain and 2OG-FeII_Oxy domain were localized in the first exon (Fig. 3A). Compared with the anthocyanidin synthesis of *A. thaliana* (AtLDOX, PDBID:1GP5), 2OGD5 lost two substrate-binding sites, which were localized at aa 141 and aa 213 in AtLDOX. Additionally, the anther substrate-binding site at aa 233 of AtLDOX exhibited a T-to-S mutation. However, the 2-oxoglutarate-binding region and iron-catalytic metal-ion-binding sites were conserved in 2OGD5 and AtLDOX. The protein domain, corresponding regions, and binding sites of 2OGD5 in the three-dimensional structure were predicted using the AtLDOX model and are shown in Fig. 3B.

### Functional analysis revealing that 2OGD5 participates in tanshinone production

**Fig. 4.**
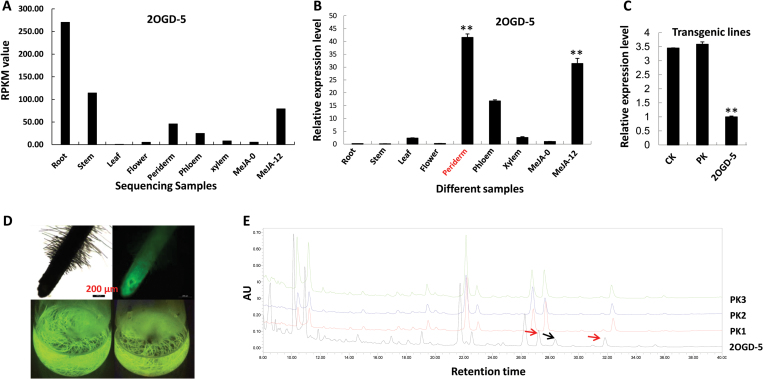
Biochemical identification of 2OGD5 in *S. miltiorrhiza*. (A) The gene expression of *2OGD5* based on the RNA-seq data in different organs, root tissues, and following MeJA treatment. MeJA-12 indicates that the leaves were sprayed with 200 μM MeJA for 12 h. (B) The *2OGD5* gene expression based on qPCR analysis in different organs, root tissues, and following MeJA treatment. (C) The expression levels of 2OGD5 in control and RNAi hairy roots. ‘CK’ is the hairy roots following infection with ACCC10060; ‘PK’ is the hairy roots following infection with ACCC10060 carried in the PK7GWIWG2D(II) vector. ‘2OGD5’ is the hairy roots following infection with ACCC10060 carried in the PK7GWIWG2D(II)-2OGD5 vector. (D) Positive green-fluorescent transgenic hairy roots expressing GFP. (E) Tanshinone variation determined using UPLC detection in the different hairy roots; AU, arbitrary units. The red arrows highlight where there is a decrease in the content of the compound relative to the control lines, and the black arrow highlights where there is an increase. In (B, C) the asterisks represent significant differences (**P<0.01) using one-way ANOVA analysis.

To determine the detailed functions of the candidate 2OGDs in *vivo*, an RNAi approach – the hairy root system – was introduced to *S. miltiorrhiza* to knockdown the expression of *2OGD5* mediated by *R. rhizogenes*. To avoid the homologous gene silencing of *2OGD5*, a genome-wide strategy was used to choose the gene fragment for specific binding of the *2OGD5* gene. A Gateway vector with GFP sequences and fragments of *2OGD5* was successfully constructed, and the positive hairy roots were first selected by detecting the GFP signal (Fig. 4). The morphology of 2OGD5-RNAi hairy roots was the same as that of hairy roots after the introduction of ACCC10060 with the PK7GWIWG2D(II) empty vector (PK). The *2OGD5* gene expression of RNAi transgenic lines was 3.6 times lower than that in the PK. After culturing for 60 d, the hairy roots and culture liquid were significantly red/brown, mainly because of the generation of tanshinones. UPLC was used to analyse the content diversity of diterpenoid compounds (Fig. 4), and the results indicated that the content of cryptotanshinone (retention time: 28 min) in the 2OGD5-RNAi lines was much lower than that in the PK, and that a new peak appeared at 29 min retention time in the 2OGD5-RNAi line. The peak area of tanshinone IIA (retention time: 32 min) also decreased in the 2OGD5-RNAi line.

**Fig. 5.**
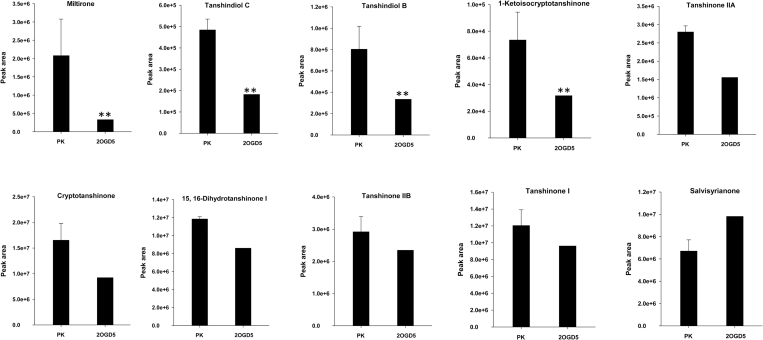
The relative quantification of tanshinones in different hairy roots based on the LC-MS/MS analysis. The error bars show the standard deviation from three independent RNAi hairy roots. Asterisks (**) represent a significant difference (more than 2-fold, *P*<0.05) using Student’s *t*-test.

To further investigate the detailed function of 2OGD5, an LC-mass spectrometry (LC-MS) system was utilized to detect the various metabolites (Fig. 5). A total of 609 metabolites were detected, and 24 tanshinones were further analysed. The relative quantification of tanshinone products indicated that 12 of the metabolites showed reduced accumulation (>1.5-fold change) in the 2OGD5-silencing lines, and four metabolites decreased significantly (>2-fold change; Supplementary Table S6), including 1-ketoisocryptotanshinone, tanshindiol B, tanshindiol C, and miltirone. The decrease of miltirone in the 2OGD5-silencing lines was the most significant, being up to 6-fold. The contents of cryptotanshinone and tanshinone IIA were found to be similar, and both were 0.56 times less than those of their control lines. The silencing of *2OGD5* led to a slight decrease in the production of dihydrotanshinone I and tanshinone I. In contrast, a slight increase (1.46-fold) in salvisyrianone was observed in the silenced *2OGD5* lines compared to the control lines.

## Discussion

### *Functional analysis of 2OGDs* in S. miltiorrhiza *provides solid evidence for its catalytic roles in tanshinone metabolism and evolutionary diversity*

Given the high oxidation activity of tanshinones, *S. miltiorrhiza* has been described as an ideal model to study the biosynthesis and regulation of diterpenoid products. Elucidating the tanshinone biosynthetic pathway has drawn the attention of many researchers, and great progress has been achieved, such as the identification of the MVA and MEP pathways, CPS and KSL diterpenoid synthases, and three CYP450s (CYP76AH1, CYP76AH3, and CYP76AK1) using a series of genomic, transcriptomic, and metabonomic technologies (Kai *et al.*, 2011; Guo *et al.*, 2013, 2016; Yang *et al.*, 2013; Luo *et al.*, 2014; Cui *et al.*, 2015); however, many unknown oxidases still need to be identified. Within the 2OGD category, many clades have been clustered and linked to catalytic activity in secondary metabolic pathways, such as GA catabolism, auxin metabolism, terpenoid indole alkaloid biosynthesis, and glucosinolate metabolism (Han and Zhu, 2011; Kawai *et al.*, 2014; Zi *et al.*, 2014). Moreover, co-operative oxidation by CYPS and 2OGDs with glycosylation by uridine diphosphate (UDP)-glucuronosyltransferases (UGTs) have been detected in some specialized metabolic pathways (Yonekura-Sakakibara and Hanada, 2011; Caputi *et al.*, 2012; Boutanaev *et al.*, 2015). However, no experimental evidence has indicated that 2OGDs participate directly in tanshinone biosynthesis. The genome-wide identification of the 2OGD superfamily will facilitate the elucidation of tanshinone biosynthesis. In examining the function of 2OGD5 in *S. miltiorrhiza* using a combination of RNAi, quantitative reverse transcriptase polymerase chain reaction (qRT-PCR), UPLC, and LC-MS, we confirmed that it plays an important role in tanshinone biosynthesis. The tanshinone contents in the *2OGD5* transgenic lines varied significantly (Fig. 5). Based on the metabolic flux of 2OGD5-RNAi, the production of downstream tanshinones integrally decreased to different extents. The increase of salvisyrianone suggested that it might be the precursor compound of miltirone. Miltirone is the group III compound of tanshinone biosynthesis and the crucial precursor in the formation of the various tanshinones (Cui *et al.*, 2015). Here, 2OGD5 was shown to contribute to miltirone biosynthesis. Previous studies have suggested a regular order in which 2OGD has an oxidation function after the hydroxylation of CYPs (Kawai *et al.*, 2014). Our results supported this proposal, indicating that 2OGD5 has an oxidation function in miltirone, cryptotanshinone, and tanshinone IIA biosynthesis. Furthermore, the extension of the 2OGD superfamily occurred after the number of CYPs increased, and the CYPs (437) are there times more abundant than 2OGDs in *S. miltiorrhiza* (Xu *et al.*, 2016*a*), indicating that the functional evolution of the DOXC clade in secondary metabolite biosynthesis followed CYP diversification. Here, the catalytic roles of 2OGD5 in tanshinone production constitute a new reference for highly oxydic diterpene biosynthesis.

### *The extension of the 2OGD superfamily members in* S. miltiorrhiza *facilitates the diversity of active compounds, especially tanshinones*

The 2OGD superfamily is widely distributed in microorganisms, fungi, plants, and mammals, and it plays a role in various oxidation reactions in widespread biological processes, such as DNA demethylation, hormone biosynthesis, and the biosynthesis of specialized secondary metabolites in response to biotic and abiotic stress. Based on previous studies of 2OGDs in other plants, such as *A. thaliana*, *O. sativa*, *Zea mays*, *Catharanthus roseus*, and *Papaver somniferum* (Kawai *et al.*, 2014), the potential catalytic role of 2OGDs in tanshinone biosynthesis appears to be reasonable. In *S. miltiorrhiza*, 132 2OGD gene members were identified, which is similar to the number in seed plants, but higher than that in lower plants, such as *Selaginella moellendorffi* (74), *Physcomitrella patens* (66), and *Chlamydomonas reinhardtii* (41). Among these 2OGD members, 118 DOXC clade members associated with biosyntheses via specialized metabolic pathways were identified, and the DOXC numbers showed significant expansion, unlike the known DOXC clades in other species, such as *A. thaliana* (96) and *O. sativa* (89). Thus, the active natural products in medicinal plants are more complex than those in some crops or model plants. These results indicated that the extension of 2OGDs promotes the accumulation and diversity of secondary metabolites to defend against environmental stresses. BLASTP and CLANS analyses revealed only three DOXA genes in *S. miltiorrhiza*, whereas the DOXA and DOXB members were indistinguishable in the phylogenetic analyses of *A. thaliana* and *O. sativa* (see Supplementary Fig. S1). The homoplasy and extremely small number of genes in the DOXA and DOXB classes in the 2OGD superfamily indicated that the evolution of 2OGDs was oriented toward oxidative activity in diverse lineage-specialized metabolisms, and that the functions related to DNA repair or proline hydroxylation of cell wall proteins were off-center. The overall amino acid sequence alignments revealed a low similarity of 2OGD superfamily members in *S. miltiorrhiza*, although the carboxyl core contained the conserved 2OG-Fell_Oxy domain. The motif analysis by MEME indicated that the evolution of some 2OGDs was significantly different for the conserved 2OGD-FeII_Oxy motifs. Based on the results of the 2OGD phylogenetic tree, the evolutionary divergence within the 2OGD superfamily in *S. miltiorrhiza* mainly occurred between the DOXB and DOXC classes. All eight 2OGDs that contained very small amounts of conserved motifs belonged to the P4H family, indicating that motifs 2, 4, 6, 1, and 9 are lineage-specific functional motifs involved in the oxidation route.

### Genome-wide identification and co-expression analysis in different organs and tissues could better reflect the potential gene function in specific secondary metabolic pathways

As an example, the accumulation of artemisinin and the high expression of key enzymatic genes in glands and trichomes of *Artemisia annua* provide a basis for investigating artemisinin biosynthesis (Maes *et al.*, 2011; Soetaert *et al.*, 2013). Our previous study of *S. miltiorrhiza* also revealed that tanshinones are produced and accumulate in the periderm of the root (Xu *et al.*, 2015). To determine the candidate 2OGDs involved in the biosynthesis of various metabolites, more than 85 Gb of transcriptome data from nine different organs and tissues were subjected to an integration study (Luo *et al.*, 2014; Xu *et al.*, 2015, 2016*a*), combined with phylogenetic-tree analysis. In *S. miltiorrhiza*, 15 2OGD genes were predicted to be members of the F3H/FLS/ANS family, which is involved in flavonoid metabolism; however, a gene number extension was observed, unlike in other known species. Two specific genes expressed in flowers were identified and predicted to contribute to anthocyanidin biosynthesis. The periderm of *S. miltiorrhiza* roots was identified as the factory of tanshinone biosynthesis and, in this tissue, the key enzymatic genes involved in tanshinone production were highly expressed. Therefore, 13 2OGDs from the DOXC class were selected according to differential expression analysis of the periderm, phloem, and xylem. The D4H/GSLOH/BX6 clade included six candidate 2OGDs. This co-expression suggested that the genes localized in the D4H/GSLOH/BX6 clade may participate in tanshinone biosynthesis. Many members of this clade are involved in different secondary metabolic pathways, such as glucosinolate, benzoxazinoid, and monoterpenoid indole alkaloid biosynthesis. However, no other evidence suggested that 2OGDs from this clade play critical roles in diterpene biosynthesis. According to the root-specific expression results, in this study, one 2OGD5 gene from the D4H/GSLOH/BX6 clade was predicted to be involved in tanshinone biosynthesis. Thus, the genetic manipulation of candidate 2OGD genes determined the effectiveness of target gene selection through the systematic use of genomic and transcriptomic tools.

## Supplementary data

Supplementary data are available at *JXB* online.

Fig. S1. Phylogenetic tree of 2OGD genes from *S. miltiorrhiza*, *A. thaliana*, and *O. sativa*.

Fig. S2. Gene structure analysis of 2OGD genes in *S. miltiorrhiza.*

Fig. S3. Conserved motif analysis of 2OGD genes in *S. miltiorrhiza*.

Fig. S4. Ten conserved motif sequences of 2OGD genes in *S. miltiorrhiza*.

Fig. S5. Gene ontology (GO) annotation analysis of 2OGD genes in *S. miltiorrhiza*.

Table S1. The primers used for qPCR analysis, full-length cloning, and gateway clones of RNAi vectors.

Table S2. The predicted cellular localization of 2OGD superfamily members in *S. miltiorrhiza*.

Table S3. The identification of homologous 2OGD members between *S. miltiorrhiza*, *A. thaliana*, and *O. sativa*.

Table S4. The gene expression of 2OGDs in different organs, tissues, and following MeJA treatment.

Table S5. Candidate 2OGDs related to tanshinone biosynthesis in *S. miltiorrhiza*.

Table S6. Different tanshinone metabolites analysed from *2OGD5*-silencing lines and control lines.

## Supplementary Material

Supplementary Figures S1-S5 and Tables S1-S6
