# Supplementary material for: The 2-oxoglutarate-dependent dioxygenase superfamily participates in tanshinone production in Salvia miltiorrhiza
Source: J Exp Bot. 2017 Apr 8;68(9):2299–308. doi: 10.1093/jxb/erx113 (PMC5447875; doi:10.1093/jxb/erx113)
Supplement: Supplementary Figures S1-S5 and Tables S1-S6 [file erx113_suppl_supplementary_figures_S1_S5_Tables_S1_S6.pdf]

Supplementary data

The 2-oxoglutarate-dependent dioxygenase superfamily participates in tanshinone production of *Salvia miltiorrhiza*

Zhichao Xu[1], Jingyuan Song[1]*
[1]Institute of Medicinal Plant Development, Chinese Academy of Medical Sciences & Peking Union Medical College, Key Laboratory of Bioactive Substances and Resources Utilization of Chinese Herbal Medicine, Ministry of Education, Beijing, China.

Fig. S1. Phylogenetic tree of 2OGD genes from *S. miltiorrhiza*, *A. thaliana*, and *O. sativa*.

Fig. S2. Gene structure analysis of 2OGD genes in *S. miltiorrhiza*.

Fig. S3. Conserved motif analysis of 2OGD genes in *S. miltiorrhiza*.

Fig. S4. Ten conserved motif sequences of 2OGD genes in *S. miltiorrhiza*.

Fig. S5. Gene ontology (GO) annotation analysis of 2OGD genes in *S. miltiorrhiza*.

Table S1. The primers used for qPCR analysis, full-length cloning, and gateway clones of RNAi vectors.

Table S2. The predicted cellular localization of 2OGD superfamily members in *S. miltiorrhiza*.

Table S3. The identification of homologous 2OGD members between *S. miltiorrhiza*, *A. thaliana*, and *O. sativa*.

Table S4. The gene expression of 2OGDs in different organs, tissues, and following MeJA treatment.

Table S5. Candidate 2OGDs related to tanshinone biosynthesis in *S. miltiorrhiza*.

Table S6. Different tanshinone metabolites analysed from 2OGD5-silencing lines and control lines.

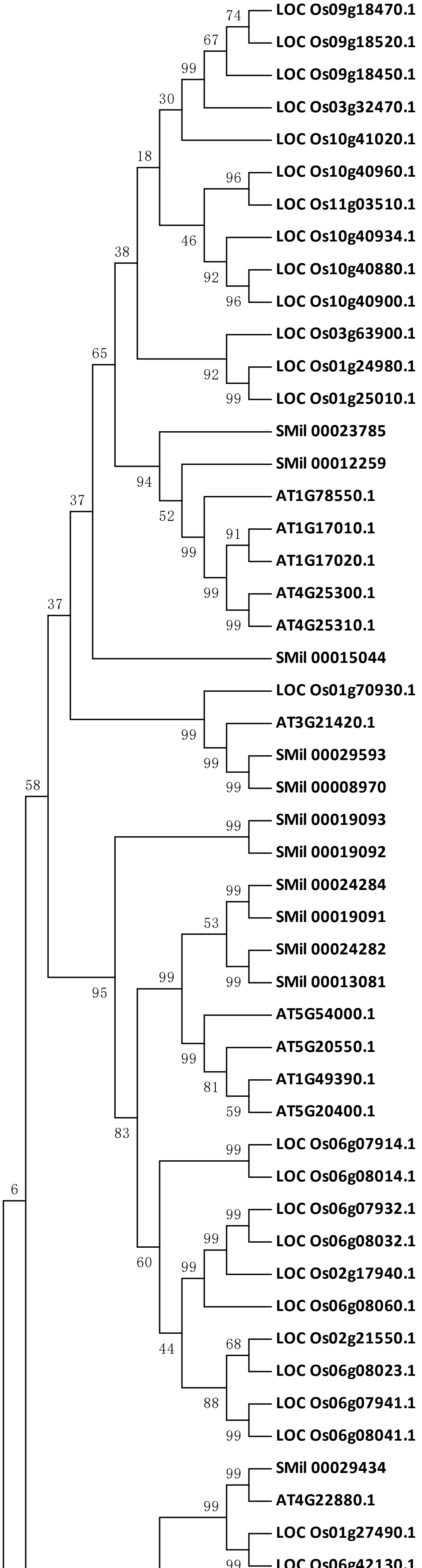
LOC Os09g18470.1
LOC Os09g18520.1
LOC Os09g18450.1
LOC Os03g32470.1
LOC Os10g41020.1
LOC Os10g40960.1
LOC Os11g03510.1
LOC Os10g40934.1
LOC Os10g40880.1
LOC Os10g40900.1
LOC Os03g63900.1
LOC Os01g24980.1
LOC Os01g25010.1
SMil 00023785
SMil 00012259
AT1G78550.1
AT1G17010.1
AT1G17020.1
AT4G25300.1
AT4G25310.1
SMil 00015044
LOC Os01g70930.1
AT3G21420.1
SMil 00029593
SMil 00008970
SMil 00019093
SMil 00019092
SMil 00024284
SMil 00019091
SMil 00024282
SMil 00013081
AT5G54000.1
AT5G20550.1
AT1G49390.1
AT5G20400.1
LOC Os06g07914.1
LOC Os06g08014.1
LOC Os06g07932.1
LOC Os06g08032.1
LOC Os02g17940.1
LOC Os06g08060.1
LOC Os02g21550.1
LOC Os06g08023.1
LOC Os06g07941.1
LOC Os06g08041.1
SMil 00029434
AT4G22880.1
LOC Os01g27490.1
LOC Os06g42130.1

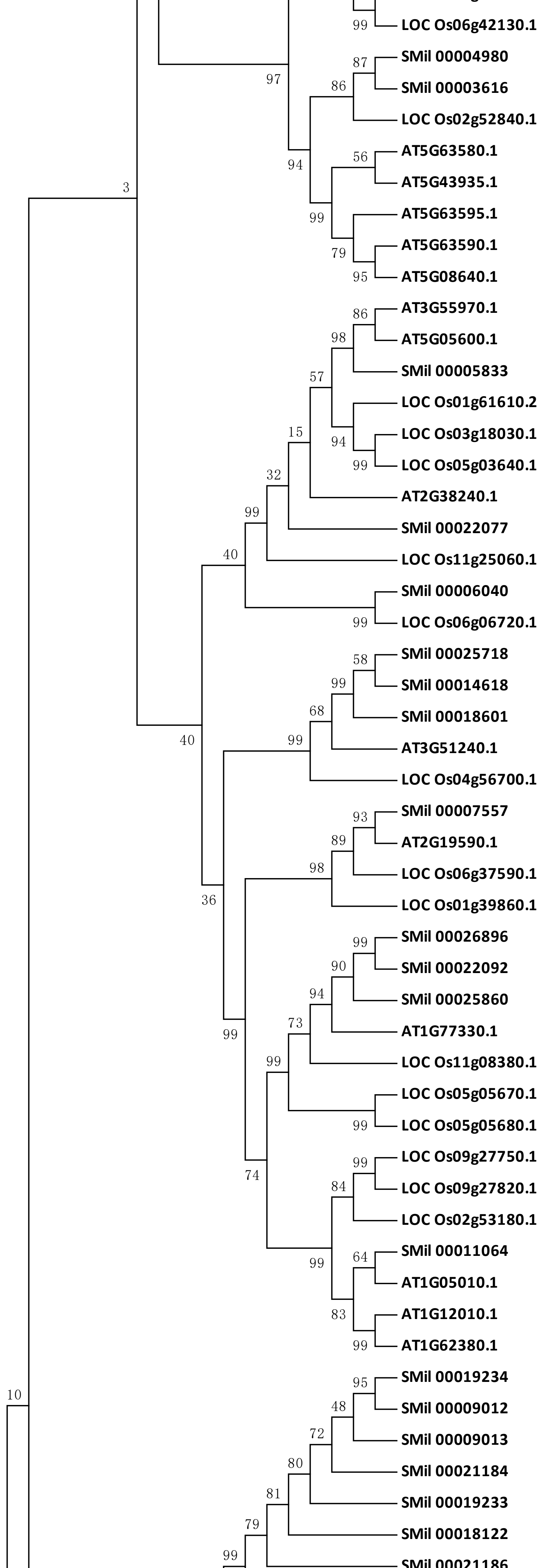
LOC Os06g42130.1
SMil 00004980
SMil 00003616
LOC Os02g52840.1
AT5G63580.1
AT5G43935.1
AT5G63595.1
AT5G63590.1
AT5G08640.1
AT3G55970.1
AT5G05600.1
SMil 00005833
LOC Os01g61610.2
LOC Os03g18030.1
LOC Os05g03640.1
AT2G38240.1
SMil 00022077
LOC Os11g25060.1
SMil 00006040
LOC Os06g06720.1
SMil 00025718
SMil 00014618
SMil 00018601
AT3G51240.1
LOC Os04g56700.1
SMil 00007557
AT2G19590.1
LOC Os06g37590.1
LOC Os01g39860.1
SMil 00026896
SMil 00022092
SMil 00025860
AT1G77330.1
LOC Os11g08380.1
LOC Os05g05670.1
LOC Os05g05680.1
LOC Os09g27750.1
LOC Os09g27820.1
LOC Os02g53180.1
SMil 00011064
AT1G05010.1
AT1G12010.1
AT1G62380.1
SMil 00019234
SMil 00009012
SMil 00009013
SMil 00021184
SMil 00019233
SMil 00018122
SMil 00021186

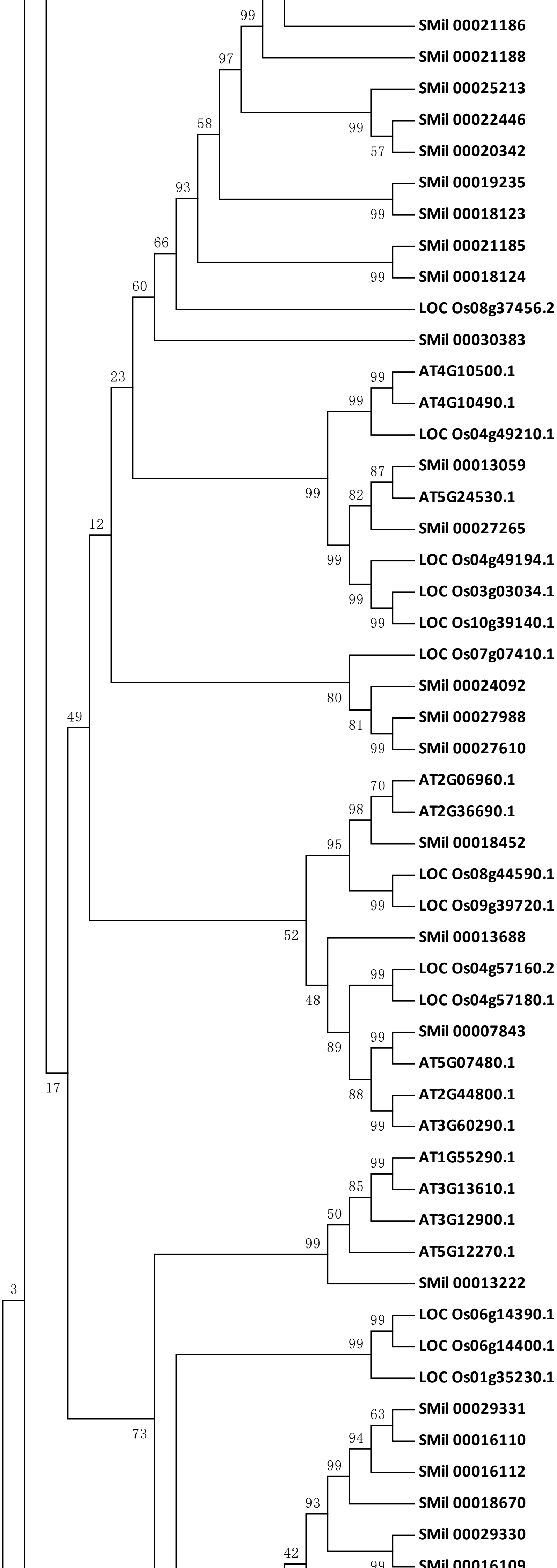
99
SMil 00021186
97
SMil 00021188
SMil 00025213
58
99
SMil 00022446
57
SMil 00020342
93
SMil 00019235
99
SMil 00018123
66
SMil 00021185
99
SMil 00018124
60
LOC Os08g37456.2
SMil 00030383
23
99
AT4G10500.1
99
AT4G10490.1
LOC Os04g49210.1
87
SMil 00013059
99
82
AT5G24530.1
12
SMil 00027265
99
LOC Os04g49194.1
99
LOC Os03g03034.1
99
LOC Os10g39140.1
LOC Os07g07410.1
SMil 00024092
80
49
SMil 00027988
81
99
SMil 00027610
70
AT2G06960.1
98
AT2G36690.1
95
SMil 00018452
LOC Os08g44590.1
99
LOC Os09g39720.1
52
SMil 00013688
99
LOC Os04g57160.2
48
LOC Os04g57180.1
99
SMil 00007843
89
AT5G07480.1
17
88
AT2G44800.1
99
AT3G60290.1
99
AT1G55290.1
85
AT3G13610.1
50
AT3G12900.1
99
AT5G12270.1
SMil 00013222
3
99
LOC Os06g14390.1
99
LOC Os06g14400.1
LOC Os01g35230.1
63
SMil 00029331
73
94
SMil 00016110
99
SMil 00016112
93
SMil 00018670
42
SMil 00029330
99
SMil 00016109

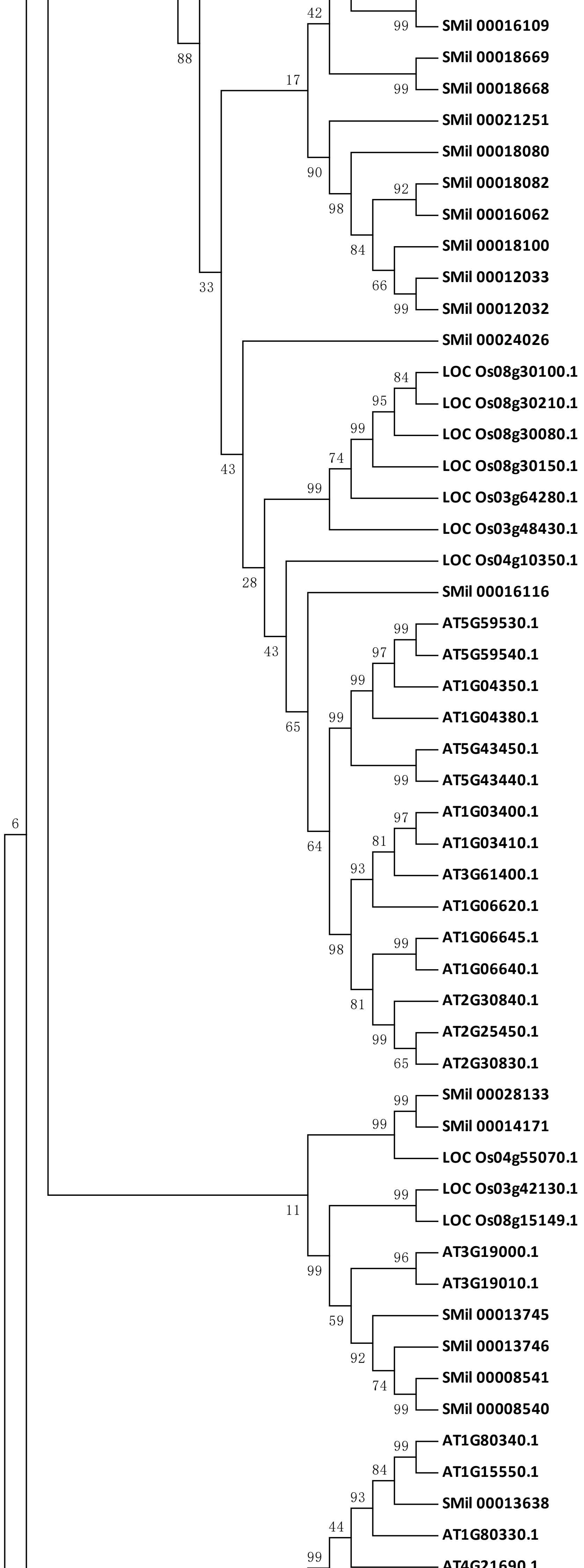
SMil 00016109
SMil 00018669
SMil 00018668
SMil 00021251
SMil 00018080
SMil 00018082
SMil 00016062
SMil 00018100
SMil 00012033
SMil 00012032
SMil 00024026
LOC Os08g30100.1
LOC Os08g30210.1
LOC Os08g30080.1
LOC Os08g30150.1
LOC Os03g64280.1
LOC Os03g48430.1
LOC Os04g10350.1
SMil 00016116
AT5G59530.1
AT5G59540.1
AT1G04350.1
AT1G04380.1
AT5G43450.1
AT5G43440.1
AT1G03400.1
AT1G03410.1
AT3G61400.1
AT1G06620.1
AT1G06645.1
AT1G06640.1
AT2G30840.1
AT2G25450.1
AT2G30830.1
SMil 00028133
SMil 00014171
LOC Os04g55070.1
LOC Os03g42130.1
LOC Os08g15149.1
AT3G19000.1
AT3G19010.1
SMil 00013745
SMil 00013746
SMil 00008541
SMil 00008540
AT1G80340.1
AT1G15550.1
SMil 00013638
AT1G80330.1

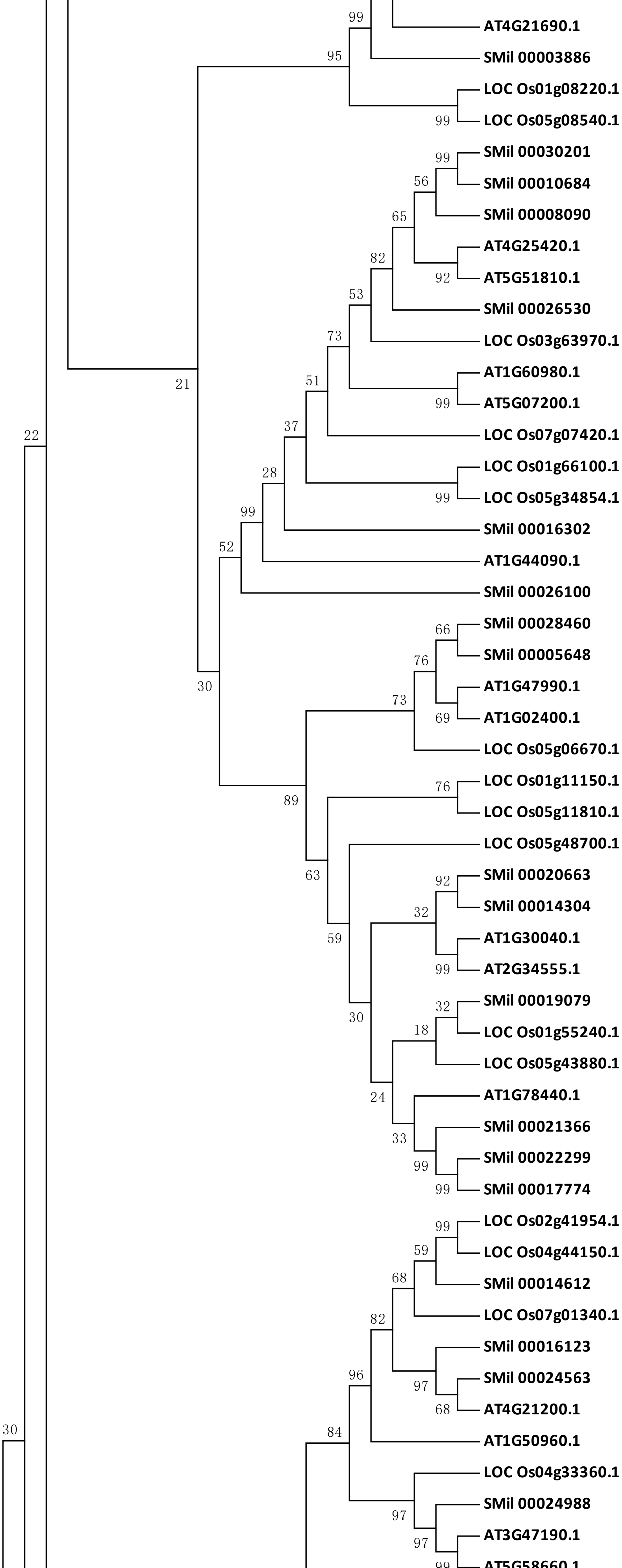
AT4G21690.1
SMil 00003886
LOC Os01g08220.1
LOC Os05g08540.1
SMil 00030201
SMil 00010684
SMil 00008090
AT4G25420.1
AT5G51810.1
SMil 00026530
LOC Os03g63970.1
AT1G60980.1
AT5G07200.1
LOC Os07g07420.1
LOC Os01g66100.1
LOC Os05g34854.1
SMil 00016302
AT1G44090.1
SMil 00026100
SMil 00028460
SMil 00005648
AT1G47990.1
AT1G02400.1
LOC Os05g06670.1
LOC Os01g11150.1
LOC Os05g11810.1
LOC Os05g48700.1
SMil 00020663
SMil 00014304
AT1G30040.1
AT2G34555.1
SMil 00019079
LOC Os01g55240.1
LOC Os05g43880.1
AT1G78440.1
SMil 00021366
SMil 00022299
SMil 00017774
LOC Os02g41954.1
LOC Os04g44150.1
SMil 00014612
LOC Os07g01340.1
SMil 00016123
SMil 00024563
AT4G21200.1
AT1G50960.1
LOC Os04g33360.1
SMil 00024988
AT3G47190.1

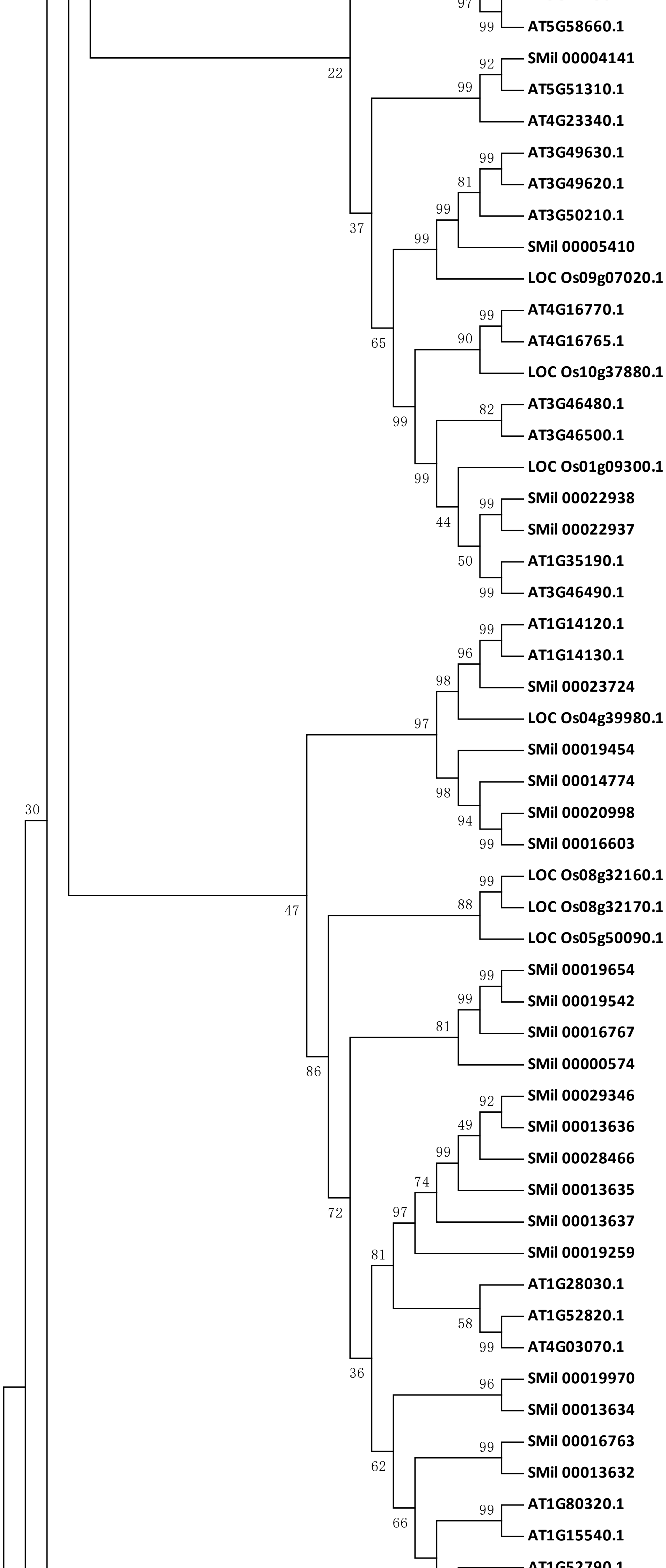
AT5G58660.1
SMil 00004141
AT5G51310.1
AT4G23340.1
AT3G49630.1
AT3G49620.1
AT3G50210.1
SMil 00005410
LOC Os09g07020.1
AT4G16770.1
AT4G16765.1
LOC Os10g37880.1
AT3G46480.1
AT3G46500.1
LOC Os01g09300.1
SMil 00022938
SMil 00022937
AT1G35190.1
AT3G46490.1
AT1G14120.1
AT1G14130.1
SMil 00023724
LOC Os04g39980.1
SMil 00019454
SMil 00014774
SMil 00020998
SMil 00016603
LOC Os08g32160.1
LOC Os08g32170.1
LOC Os05g50090.1
SMil 00019654
SMil 00019542
SMil 00016767
SMil 00000574
SMil 00029346
SMil 00013636
SMil 00028466
SMil 00013635
SMil 00013637
SMil 00019259
AT1G28030.1
AT1G52820.1
AT4G03070.1
SMil 00019970
SMil 00013634
SMil 00016763
SMil 00013632
AT1G80320.1
AT1G15540.1
AT1G52790.1

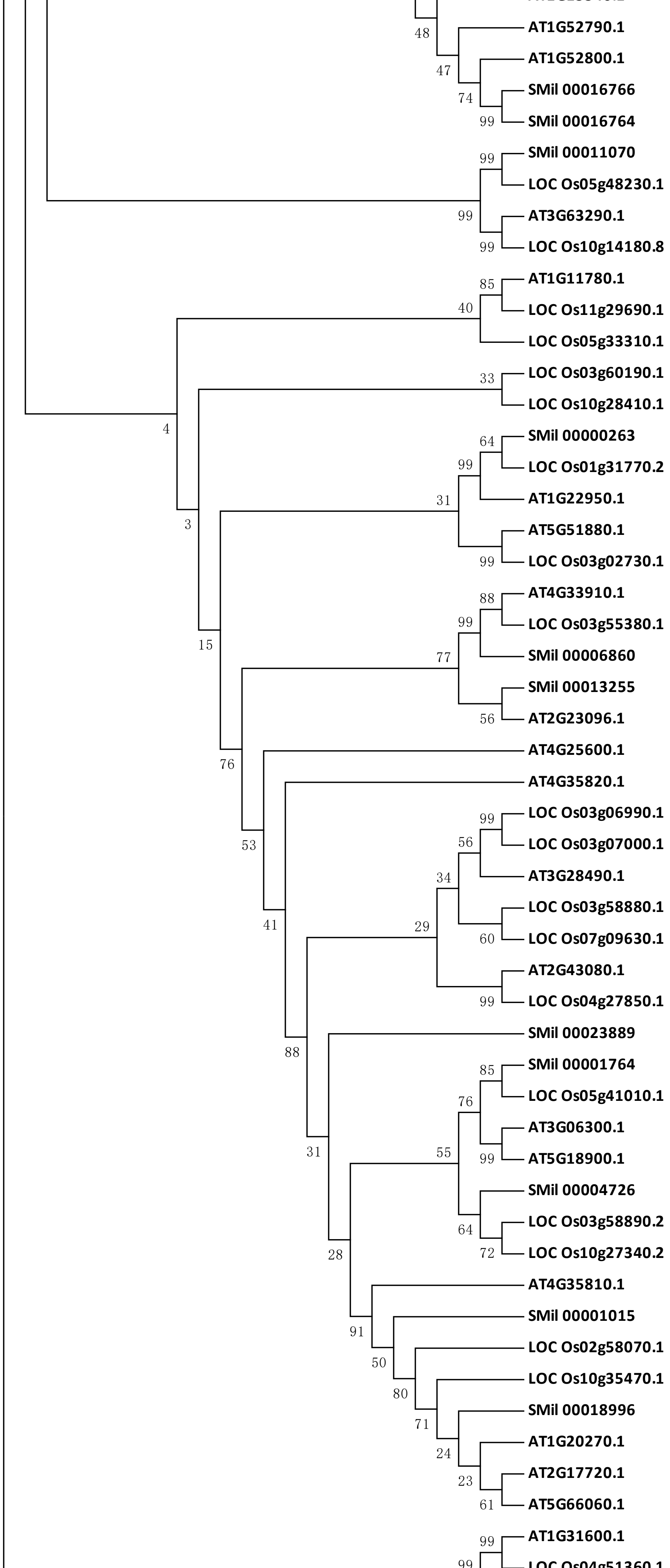
AT1G52790.1
AT1G52800.1
SMil 00016766
SMil 00016764
SMil 00011070
LOC Os05g48230.1
AT3G63290.1
LOC Os10g14180.8
AT1G11780.1
LOC Os11g29690.1
LOC Os05g33310.1
LOC Os03g60190.1
LOC Os10g28410.1
SMil 00000263
LOC Os01g31770.2
AT1G22950.1
AT5G51880.1
LOC Os03g02730.1
AT4G33910.1
LOC Os03g55380.1
SMil 00006860
SMil 00013255
AT2G23096.1
AT4G25600.1
AT4G35820.1
LOC Os03g06990.1
LOC Os03g07000.1
AT3G28490.1
LOC Os03g58880.1
LOC Os07g09630.1
AT2G43080.1
LOC Os04g27850.1
SMil 00023889
SMil 00001764
LOC Os05g41010.1
AT3G06300.1
AT5G18900.1
SMil 00004726
LOC Os03g58890.2
LOC Os10g27340.2
AT4G35810.1
SMil 00001015
LOC Os02g58070.1
LOC Os10g35470.1
SMil 00018996
AT1G20270.1
AT2G17720.1
AT5G66060.1
AT1G31600.1

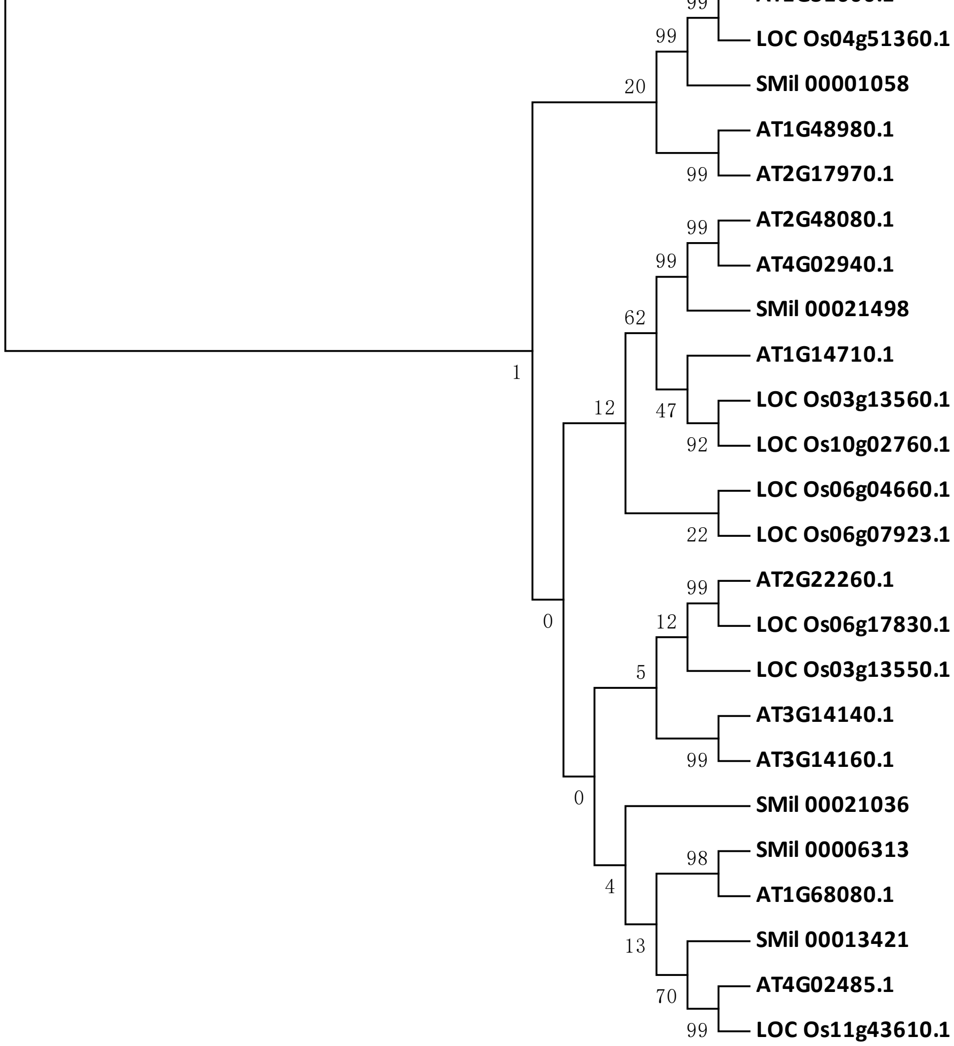
LOC Os04g51360.1
SMil 00001058
AT1G48980.1
AT2G17970.1
AT2G48080.1
AT4G02940.1
SMil 00021498
AT1G14710.1
LOC Os03g13560.1
LOC Os10g02760.1
LOC Os06g04660.1
LOC Os06g07923.1
AT2G22260.1
LOC Os06g17830.1
LOC Os03g13550.1
AT3G14140.1
AT3G14160.1
SMil 00021036
SMil 00006313
AT1G68080.1
SMil 00013421
AT4G02485.1
LOC Os11g43610.1

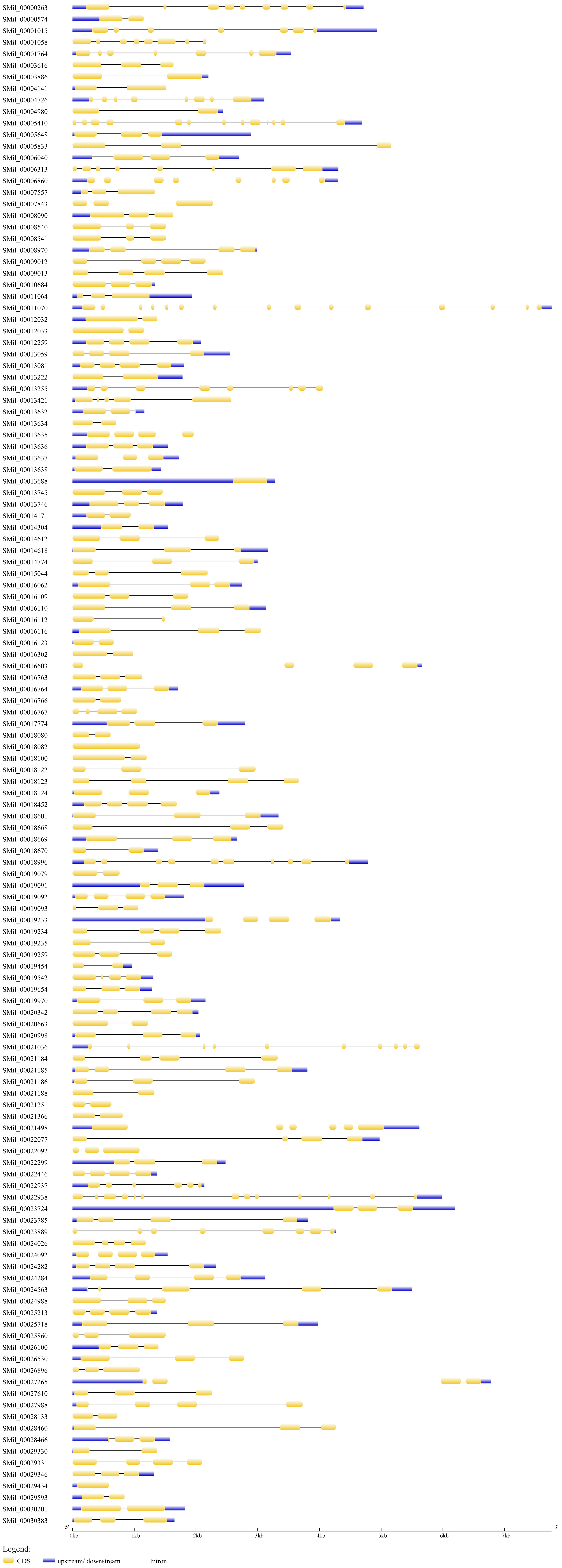

SMil_00000263
SMil_00000574
SMil_00001015
SMil_00001058
SMil_00001764
SMil_00003616
SMil_00003886
SMil_00004141
SMil_00004726
SMil_00004980
SMil_00005410
SMil_00005648
SMil_00005833
SMil_00006040
SMil_00006313
SMil_00006860
SMil_00007557
SMil_00007843
SMil_00008090
SMil_00008540
SMil_00008541
SMil_00008970
SMil_00009012
SMil_00009013
SMil_00010684
SMil_00011064
SMil_00011070
SMil_00012032
SMil_00012033
SMil_00012259
SMil_00013059
SMil_00013081
SMil_00013222
SMil_00013255
SMil_00013421
SMil_00013632
SMil_00013634
SMil_00013635
SMil_00013636
SMil_00013637
SMil_00013638
SMil_00013688
SMil_00013745
SMil_00013746
SMil_00014171
SMil_00014304
SMil_00014612
SMil_00014618
SMil_00014774
SMil_00015044
SMil_00016062
SMil_00016109
SMil_00016110
SMil_00016112
SMil_00016116
SMil_00016123
SMil_00016302
SMil_00016603
SMil_00016763
SMil_00016764
SMil_00016766
SMil_00016767
SMil_00017774
SMil_00018080
SMil_00018082
SMil_00018100
SMil_00018122
SMil_00018123
SMil_00018124
SMil_00018452
SMil_00018601
SMil_00018668
SMil_00018669
SMil_00018670
SMil_00018996
SMil_00019079
SMil_00019091
SMil_00019092
SMil_00019093
SMil_00019233
SMil_00019234
SMil_00019235
SMil_00019259
SMil_00019454
SMil_00019542
SMil_00019654
SMil_00019970
SMil_00020342
SMil_00020663
SMil_00020998
SMil_00021036
SMil_00021184
SMil_00021185
SMil_00021186
SMil_00021188
SMil_00021251
SMil_00021366
SMil_00021498
SMil_00022077
SMil_00022092
SMil_00022299
SMil_00022446
SMil_00022937
SMil_00022938
SMil_00023724
SMil_00023785
SMil_00023889
SMil_00024026
SMil_00024092
SMil_00024282
SMil_00024284
SMil_00024563
SMil_00024988
SMil_00025213
SMil_00025718
SMil_00025860
SMil_00026100
SMil_00026530
SMil_00026896
SMil_00027265
SMil_00027610
SMil_00027988
SMil_00028133
SMil_00028460
SMil_00028466
SMil_00029330
SMil_00029331
SMil_00029346
SMil_00029434
SMil_00029593
SMil_00030201
SMil_00030383
5'
0kb
1kb
2kb
3kb
4kb
5kb
6kb
7kb
3'
Legend:
CDS
upstream/ downstream
Intron

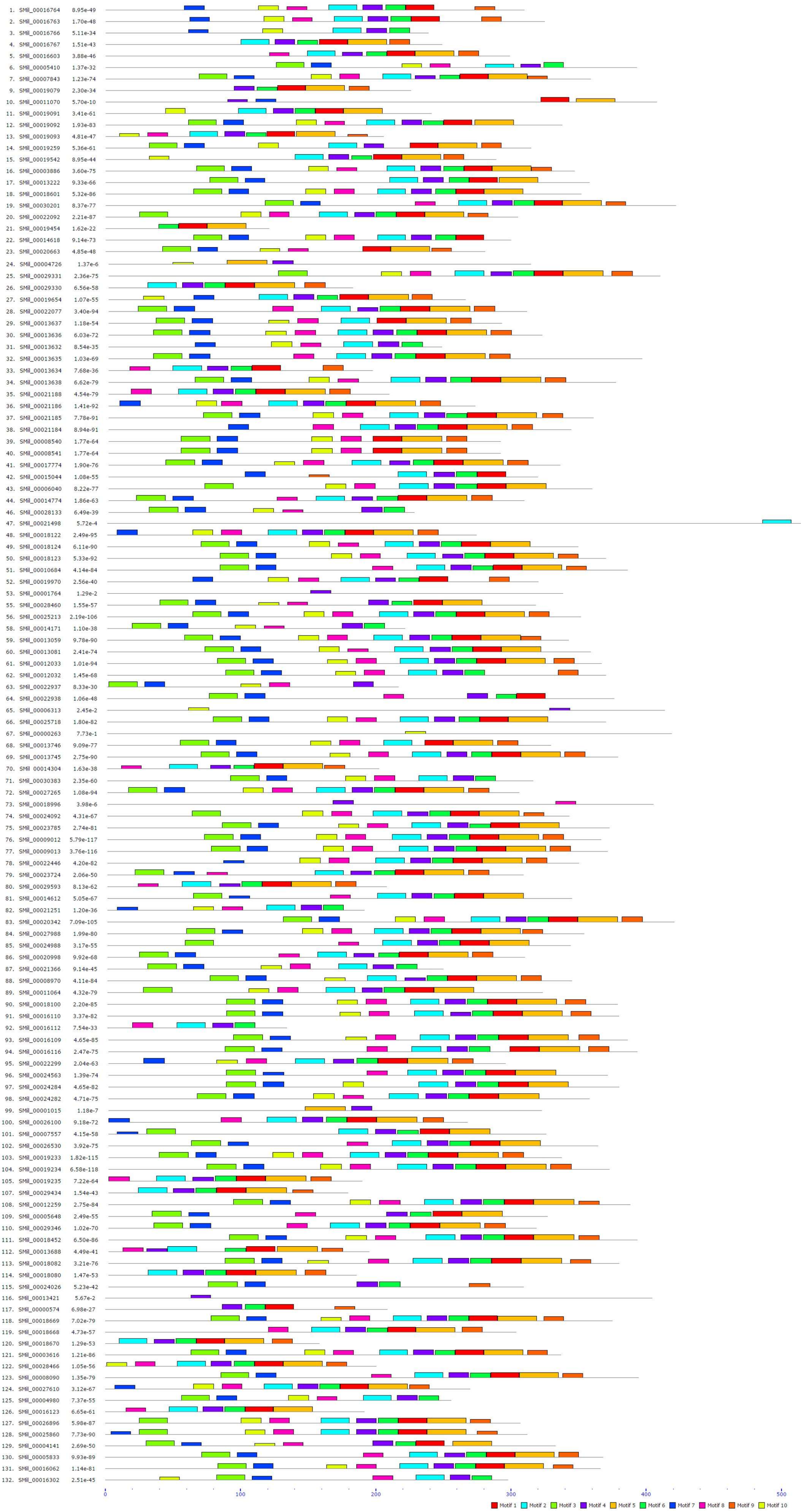

1. SMil_00016764 8.95e-49
2. SMil_00016763 1.70e-48
3. SMil_00016766 5.11e-34
4. SMil_00016767 1.51e-43
5. SMil_00016603 3.88e-46
6. SMil_00005410 1.37e-32
7. SMil_00007843 1.23e-74
9. SMil_00019079 2.30e-34
10. SMil_00011070 5.70e-10
11. SMil_00019091 3.41e-61
12. SMil_00019092 1.93e-83
13. SMil_00019093 4.81e-47
14. SMil_00019259 5.36e-61
15. SMil_00019542 8.95e-44
16. SMil_00003886 3.60e-75
17. SMil_00013222 9.33e-66
18. SMil_00018601 5.32e-86
19. SMil_00030201 8.37e-77
20. SMil_00022092 2.21e-87
21. SMil_00019454 1.62e-22
22. SMil_00014618 9.14e-73
23. SMil_00020663 4.85e-48
24. SMil_00004726 1.37e-6
25. SMil_00029331 2.36e-75
26. SMil_00029330 6.56e-58
27. SMil_00019654 1.07e-55
28. SMil_00022077 3.40e-94
29. SMil_00013637 1.18e-54
30. SMil_00013636 6.03e-72
31. SMil_00013632 8.54e-35
32. SMil_00013635 1.03e-69
33. SMil_00013634 7.68e-36
34. SMil_00013638 6.62e-79
35. SMil_00021188 4.54e-79
36. SMil_00021186 1.41e-92
37. SMil_00021185 7.78e-91
38. SMil_00021184 8.94e-91
39. SMil_00008540 1.77e-64
40. SMil_00008541 1.77e-64
41. SMil_00017774 1.90e-76
42. SMil_00015044 1.08e-55
43. SMil_00006040 8.22e-77
44. SMil_00014774 1.86e-63
46. SMil_00028133 6.49e-39
47. SMil_00021498 5.72e-4
48. SMil_00018122 2.49e-95
49. SMil_00018124 6.11e-90
50. SMil_00018123 5.33e-92
51. SMil_00010684 4.14e-84
52. SMil_00019970 2.56e-40
53. SMil_00001764 1.29e-2
55. SMil_00028460 1.55e-57
56. SMil_00025213 2.19e-106
58. SMil_00014171 1.10e-38
59. SMil_00013059 9.78e-90
60. SMil_00013081 2.41e-74
61. SMil_00012033 1.01e-94
62. SMil_00012032 1.45e-68
63. SMil_00022937 8.33e-30
64. SMil_00022938 1.06e-48
65. SMil_00006313 2.45e-2
66. SMil_00025718 1.80e-82
67. SMil_00000263 7.73e-1
68. SMil_00013746 9.09e-77
69. SMil_00013745 2.75e-90
70. SMil 00014304 1.63e-38
71. SMil_00030383 2.35e-60
72. SMil_00027265 1.08e-94
73. SMil_00018996 3.98e-6
74. SMil_00024092 4.31e-67
75. SMil_00023785 2.74e-81
76. SMil_00009012 5.79e-117
77. SMil_00009013 3.76e-116
78. SMil_00022446 4.20e-82
79. SMil_00023724 2.06e-50
80. SMil_00029593 8.13e-62
81. SMil_00014612 5.05e-67
82. SMil_00021251 1.20e-36
83. SMil_00020342 7.09e-105
84. SMil_00027988 1.99e-80
85. SMil_00024988 3.17e-55
86. SMil_00020998 9.92e-68
87. SMil_00021366 9.14e-45
88. SMil_00008970 4.11e-84
89. SMil_00011064 4.32e-79
90. SMil_00018100 2.20e-85
91. SMil_00016110 3.37e-82
92. SMil_00016112 7.54e-33
93. SMil_00016109 4.65e-85
94. SMil_00016116 2.47e-75
95. SMil_00022299 2.04e-63
96. SMil_00024563 1.39e-74
97. SMil_00024284 4.65e-82
98. SMil_00024282 4.71e-75
99. SMil_00001015 1.18e-7
100. SMil_00026100 9.18e-72
101. SMil_00007557 4.15e-58
102. SMil_00026530 3.92e-75
103. SMil_00019233 1.82e-115
104. SMil_00019234 6.58e-118
105. SMil_00019235 7.22e-64
107. SMil_00029434 1.54e-43
108. SMil_00012259 2.75e-84
109. SMil_00005648 2.49e-55
110. SMil_00029346 1.02e-70
111. SMil_00018452 6.50e-86
112. SMil_00013688 4.49e-41
113. SMil_00018082 3.21e-76
114. SMil_00018080 1.47e-53
115. SMil_00024026 5.23e-42
116. SMil_00013421 5.67e-2
117. SMil_00000574 6.98e-27
118. SMil_00018669 7.02e-79
119. SMil_00018668 4.73e-57
120. SMil_00018670 1.29e-53
121. SMil_00003616 1.21e-86
122. SMil_00028466 1.05e-56
123. SMil_00008090 1.35e-79
124. SMil_00027610 3.12e-67
125. SMil_00004980 7.37e-55
126. SMil_00016123 6.65e-61
127. SMil_00026896 5.98e-87
128. SMil_00025860 7.73e-90
129. SMil_00004141 2.69e-50
130. SMil_00005833 9.93e-89
131. SMil_00016062 1.14e-81
132. SMil_00016302 2.51e-45
0
100
200
300
400
500
Motif 1
Motif 2
Motif 3
Motif 4
Motif 5
Motif 6
Motif 7
Motif 8
Motif 9
Motif 10

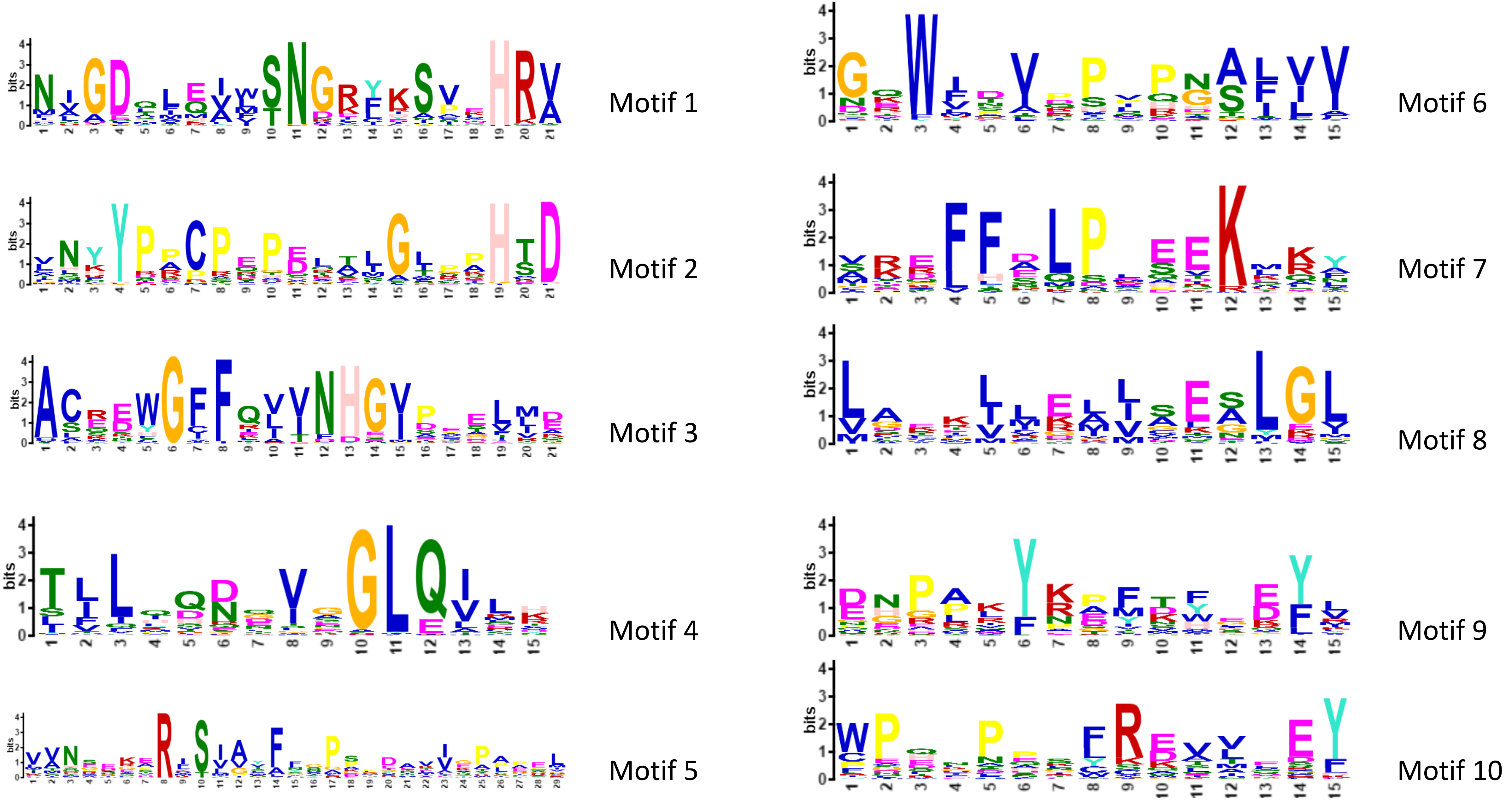
Motif 1
Motif 2
Motif 3
Motif 4
Motif 5
Motif 6
Motif 7
Motif 8
Motif 9
Motif 10

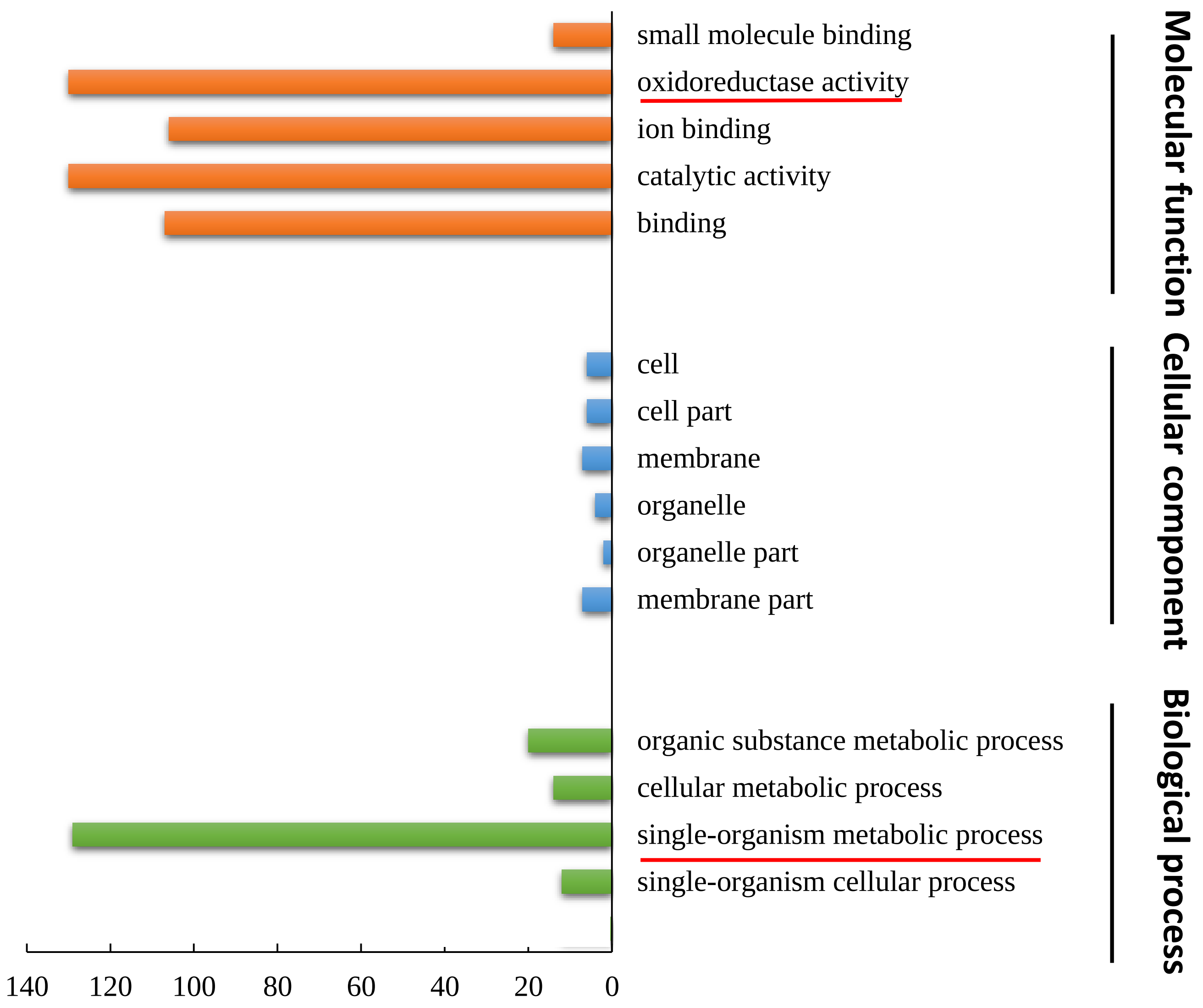
small molecule binding
oxidoreductase activity
ion binding
catalytic activity
binding
cell
cell part
membrane
organelle
organelle part
membrane part
organic substance metabolic process
cellular metabolic process
single-organism metabolic process
single-organism cellular process
140
120
100
80
60
40
20
0
Molecular function
Cellular component
Biological process

Supplementary Table1. The primers used for qPCR analysis, cloning full-length, and gateway clone of RNAi vectors.

**Gene_name**	**Use**	**Primer pairs (5'-3' Forward/Reverse)**
2OGD-5	qPCR	CGTGTCTCTATTACCCAGTCTG/CATTGACCGCCGTGAAGAA
SmActin	qPCR	AGGAACCACCGATCCAGACA/GGTGCCCTGAGGTCCTGTT
2OGD-5	Full-length	ATGGTGCTCTCTAGCAACGGTG/TCAAATCTTGTAATGAGGCAAAGCCG
2OGD-5	RNAi	GGGGACAAGTTTGTACAAAAAAGCAGGCTCCCTCGGAGACGATGGACG/ GGGGACCACTTTGTACAAGAAAGCTGGGTGTCGTCGCTGAAGACGCAC
PK-GFP	RNAi	CTCGTTGGGGTCTTTGCTC/ CACAAGTTCAGCGTGTCCG

Supplementary Table 2 The cellular localization prediction of 2OGD superfamily members in *S. miltiorrhiza*.

Name	Len	cTP	mTP	SP	other	Loc	RC
SMil_00030383	307	0.075	0.084	0.070	0.946	_	1
SMil_00030201	421	0.416	0.228	0.041	0.210	*	5
SMil_00029593	201	0.137	0.060	0.206	0.848	_	2
SMil_00029434	170	0.100	0.070	0.088	0.887	_	2
SMil_00029346	304	0.267	0.074	0.161	0.749	_	3
SMil_00029331	396	0.118	0.095	0.065	0.863	_	2
SMil_00029330	175	0.084	0.343	0.115	0.597	_	4
SMil_00028466	199	0.267	0.081	0.103	0.755	_	3
SMil_00028460	311	0.089	0.091	0.134	0.908	_	2
SMil_00028133	220	0.044	0.084	0.066	0.978	_	1
SMil_00027988	344	0.162	0.087	0.042	0.850	_	2
SMil_00027610	268	0.071	0.097	0.139	0.893	_	2
SMil_00027265	297	0.056	0.183	0.152	0.712	_	3
SMil_00026896	305	0.098	0.101	0.142	0.856	_	2
SMil_00026530	348	0.230	0.071	0.075	0.723	_	3
SMil_00026100	255	0.067	0.549	0.008	0.669	_	5
SMil_00025860	310	0.116	0.075	0.153	0.758	_	2
SMil_00025718	362	0.069	0.022	0.793	0.402	S	4
SMil_00025213	344	0.837	0.031	0.065	0.136	C	2
SMil_00024988	334	0.785	0.034	0.082	0.272	C	3
SMil_00024563	355	0.051	0.119	0.224	0.616	_	4
SMil_00024284	363	0.177	0.069	0.277	0.635	_	4
SMil_00024282	342	0.210	0.052	0.163	0.553	_	4
SMil_00024092	333	0.491	0.052	0.076	0.718	_	4
SMil_00024026	295	0.115	0.124	0.093	0.896	_	2
SMil_00023889	306	0.295	0.051	0.162	0.688	_	4
SMil_00023785	362	0.013	0.016	0.898	0.541	S	4
SMil_00023724	300	0.018	0.018	0.897	0.250	S	2
SMil_00022938	368	0.086	0.050	0.021	0.879	_	2
SMil_00022937	211	0.138	0.083	0.294	0.685	_	4
SMil_00022446	340	0.780	0.024	0.078	0.145	C	2
SMil_00022299	282	0.052	0.905	0.026	0.275	M	2
SMil_00022092	304	0.108	0.100	0.134	0.854	_	2
SMil_00022077	300	0.064	0.605	0.014	0.546	*	5
SMil_00021498	504	0.006	0.602	0.110	0.114	*	3
SMil_00021366	242	0.068	0.055	0.508	0.611	_	5
SMil_00021251	185	0.050	0.483	0.049	0.781	_	4
SMil_00021188	201	0.027	0.132	0.436	0.343	S	5
SMil_00021186	263	0.174	0.072	0.150	0.769	_	3

SMil_00021185	348	0.102	0.046	0.272	0.355	*	5
SMil_00021184	332	0.930	0.023	0.066	0.181	C	2
SMil_00021036	212	0.006	0.059	0.980	0.012	S	1
SMil_00020998	301	0.127	0.052	0.257	0.822	_	3
SMil_00020663	280	0.037	0.124	0.207	0.779	_	3
SMil_00020342	409	0.178	0.379	0.011	0.079	*	4
SMil_00019970	313	0.516	0.134	0.053	0.551	_	5
SMil_00019654	256	0.120	0.068	0.107	0.932	_	1
SMil_00019542	288	0.023	0.116	0.027	0.929	_	1
SMil_00019454	120	0.079	0.212	0.064	0.558	_	4
SMil_00019259	314	0.169	0.104	0.110	0.836	_	2
SMil_00019235	180	0.054	0.109	0.094	0.885	_	2
SMil_00019234	356	0.368	0.064	0.082	0.582	_	4
SMil_00019233	322	0.129	0.053	0.161	0.782	_	2
SMil_00019093	205	0.020	0.863	0.050	0.183	M	2
SMil_00019092	337	0.148	0.134	0.039	0.820	_	2
SMil_00019091	240	0.054	0.161	0.045	0.904	_	2
SMil_00019079	225	0.107	0.093	0.047	0.925	_	1
SMil_00018996	394	0.009	0.740	0.351	0.055	*	4
SMil_00018670	157	0.036	0.072	0.037	0.957	_	1
SMil_00018669	373	0.101	0.137	0.101	0.865	_	2
SMil_00018668	302	0.016	0.614	0.034	0.633	_	5
SMil_00018601	351	0.068	0.116	0.307	0.737	_	3
SMil_00018452	376	0.137	0.136	0.040	0.843	_	2
SMil_00018124	342	0.763	0.304	0.039	0.088	C	3
SMil_00018123	362	0.683	0.027	0.297	0.425	C	4
SMil_00018122	268	0.072	0.064	0.266	0.865	_	3
SMil_00018100	368	0.081	0.078	0.085	0.931	_	1
SMil_00018082	363	0.108	0.099	0.080	0.904	_	2
SMil_00018080	176	0.182	0.120	0.101	0.665	_	3
SMil_00017774	324	0.180	0.041	0.252	0.474	*	4
SMil_00016767	248	0.260	0.030	0.024	0.871	_	2
SMil_00016766	238	0.246	0.069	0.127	0.840	_	3
SMil_00016764	309	0.144	0.092	0.101	0.874	_	2
SMil_00016763	324	0.240	0.222	0.058	0.599	_	4
SMil_00016603	298	0.031	0.390	0.056	0.854	_	3
SMil_00016302	296	0.158	0.079	0.198	0.656	_	3
SMil_00016123	190	0.029	0.019	0.719	0.421	S	4
SMil_00016116	376	0.094	0.122	0.078	0.859	_	2
SMil_00016112	129	0.014	0.349	0.255	0.695	_	4
SMil_00016110	369	0.077	0.114	0.118	0.911	_	2
SMil_00016109	369	0.345	0.081	0.061	0.758	_	3
SMil_00016062	364	0.106	0.090	0.083	0.917	_	1
SMil_00015044	308	0.112	0.151	0.073	0.856	_	2

SMil_00014774	298	0.166	0.088	0.180	0.798	_	2
SMil_00014618	299	0.068	0.116	0.307	0.737	_	3
SMil_00014612	335	0.105	0.237	0.028	0.692	_	3
SMil_00014304	197	0.054	0.104	0.248	0.649	_	3
SMil_00014171	216	0.074	0.070	0.093	0.953	_	1
SMil_00013746	322	0.159	0.088	0.081	0.888	_	2
SMil_00013745	371	0.059	0.243	0.052	0.794	_	3
SMil_00013688	185	0.029	0.049	0.652	0.372	S	4
SMil_00013638	364	0.118	0.106	0.134	0.831	_	2
SMil_00013637	282	0.190	0.077	0.104	0.850	_	2
SMil_00013636	311	0.263	0.074	0.159	0.751	_	3
SMil_00013635	293	0.050	0.093	0.020	0.940	_	1
SMil_00013634	189	0.085	0.249	0.095	0.778	_	3
SMil_00013632	239	0.381	0.099	0.109	0.609	_	4
SMil_00013421	428	0.122	0.177	0.088	0.677	_	3
SMil_00013255	327	0.002	0.480	0.677	0.249	S	5
SMil_00013222	357	0.122	0.103	0.048	0.873	_	2
SMil_00013081	351	0.197	0.044	0.358	0.474	*	5
SMil_00013059	335	0.185	0.282	0.069	0.312	*	5
SMil_00012259	371	0.303	0.150	0.013	0.739	_	3
SMil_00012033	359	0.175	0.075	0.072	0.843	_	2
SMil_00012032	362	0.112	0.079	0.063	0.896	_	2
SMil_00011070	407	0.635	0.633	0.004	0.021	C	5
SMil_00011064	314	0.078	0.065	0.291	0.747	_	3
SMil_00010684	378	0.400	0.072	0.057	0.656	_	4
SMil_00009013	361	0.605	0.046	0.154	0.223	*	4
SMil_00009012	356	0.368	0.064	0.082	0.582	_	4
SMil_00008970	335	0.225	0.158	0.080	0.706	_	3
SMil_00008541	281	0.134	0.093	0.083	0.802	_	2
SMil_00008540	281	0.158	0.086	0.092	0.764	_	2
SMil_00008090	392	0.732	0.061	0.076	0.377	C	4
SMil_00007843	358	0.094	0.086	0.083	0.898	_	1
SMil_00007557	311	0.061	0.123	0.153	0.883	_	2
SMil_00006860	293	0.003	0.153	0.704	0.207	S	3
SMil_00006313	405	0.144	0.483	0.004	0.155	*	4
SMil_00006040	347	0.092	0.169	0.129	0.704	_	3
SMil_00005833	366	0.231	0.106	0.086	0.745	_	3
SMil_00005648	312	0.057	0.111	0.115	0.914	_	2
SMil_00005410	392	0.021	0.497	0.106	0.135	*	4
SMil_00004980	254	0.075	0.075	0.316	0.583	_	4
SMil_00004726	303	0.124	0.062	0.682	0.038	S	3
SMil_00004141	331	0.091	0.062	0.087	0.768	_	2
SMil_00003886	346	0.096	0.138	0.068	0.743	_	2
SMil_00003616	335	0.120	0.177	0.209	0.461	*	4

SMil_00001764	331	0.078	0.080	0.009	0.954	_	1
SMil_00001058	367	0.022	0.229	0.034	0.952	_	2
SMil_00001015	308	0.580	0.095	0.070	0.087	*	3
SMil_00000574	207	0.045	0.044	0.874	0.069	S	1
SMil_00000263	410	0.090	0.192	0.045	0.702	_	3

Supplementary Table 3. The identification of homologous 2OGD members between *S. miltiorrhiza, A. thaliana, and O. sativa*.

Sm2OGD_ID	At2OGD_ID	Class (*A. thaliana*)	Identity (*A. thaliana*)	E value (*A. thaliana*)	Bit score (*A. thaliana*)	Os2OGD_ID	Class (*O. sativa*)	Identity (*O. sativa*)	E value (*O. sativa*)	Bit score (*O. sativa*)
SMil_00001058	AT1G31600.1	DOXA	57.33	1.00E-144	410	Os04g51360	DOXA	55.18	2E-139	393
SMil_00013421	AT4G02485.1	DOXA	47.9	3.00E-31	112	Os11g43610	DOXA	43.85	3E-27	102
SMil_00021498	AT4G02940.1	DOXA	49.81	2.00E-141	412	Os05g33310	DOXA	48.49	7E-97	291
SMil_00001015	AT5G66060.1	DOXB	72.16	3.00E-152	421	Os02g58070	DOXB	75.68	3E-130	366
SMil_00001764	AT5G18900.1	DOXB	72.86	2.00E-150	418	Os05g41010	DOXB	67.54	5E-138	387
SMil_00004726	AT3G28480.1	DOXB	68.14	3.00E-162	447	Os10g27340	DOXB	69.78	2E-144	402
SMil_00006860	AT4G33910.1	DOXB	71.77	1.00E-139	389	Os03g55380	DOXB	74.22	1E-129	364
SMil_00013255	AT4G33910.1	DOXB	65.03	7.00E-135	378	Os03g55380	DOXB	58.73	8E-125	353
SMil_00018996	AT1G20270.1	DOXB	72.6	2.00E-141	397	Os02g58070	DOXB	82.43	7E-135	381
SMil_00021036	AT2G43080.1	DOXB	65.44	8.00E-104	294	Os04g27850	DOXB	65.57	1E-103	293
SMil_00023889	AT1G20270.1	DOXB	60.92	2.00E-122	345	Os02g58070	DOXB	64.14	1E-111	319
SMil_00000574	AT1G52800.1	DOXC	51.08	1.00E-69	208	Os08g32170	DOXC	31.09	4E-31	108
SMil_00003616	AT5G08640.1	DOXC	62.99	1.00E-132	375	Os02g52840	DOXC	56.42	5E-110	317
SMil_00003886	AT1G15550.1	DOXC	45.76	3.00E-103	301	Os01g08220	DOXC	39.37	5E-67	208
SMil_00004141	AT4G23340.1	DOXC	55	2.00E-108	312	Os03g63970	DOXC	31.21	7E-28	104
SMil_00004980	AT5G08640.1	DOXC	56.87	4.00E-98	283	Os02g52840	DOXC	50.38	5E-84	247
SMil_00005410	AT3G50210.1	DOXC	58.68	2.00E-142	402	Os09g07020	DOXC	56.46	5E-134	384
SMil_00005648	AT1G02400.1	DOXC	52.06	6.00E-103	298	Os05g06670	DOXC	42.26	9E-75	227
SMil_00005833	AT5G05600.1	DOXC	60.61	2.00E-162	452	Os05g03640	DOXC	55.71	4E-140	396
SMil_00006040	AT4G16330.1	DOXC	65.37	2.00E-127	358	Os06g06720	DOXC	57.76	3E-134	379

SMil_00007557	AT2G19590.1	DOXC	58.61	9.00E-134	375	Os06g37590	DOXC	48.5	3E-109	312
SMil_00007843	AT5G07480.1	DOXC	54.78	4.00E-139	393	Os04g57180	DOXC	44.12	4E-94	277
SMil_00008090	AT5G51810.1	DOXC	59.95	1.00E-162	455	Os03g63970	DOXC	56.2	1E-142	404
SMil_00008540	AT3G19010.1	DOXC	53.54	6.00E-55	174	Os08g15149	DOXC	47.12	2E-81	240
SMil_00008541	AT3G19010.1	DOXC	53.54	6.00E-55	174	Os08g15149	DOXC	47.12	3E-81	240
SMil_00008970	AT3G21420.1	DOXC	65.75	3.00E-165	458	Os01g70930	DOXC	58.47	6E-148	414
SMil_00009012	AT4G10500.1	DOXC	37.94	5.00E-65	203	Os08g37456	DOXC	43.23	2E-88	263
SMil_00009013	AT4G10490.1	DOXC	38.59	2.00E-64	201	Os08g37456	DOXC	43.91	4E-91	270
SMil_00010684	AT4G25420.1	DOXC	71.26	0	530	Os03g63970	DOXC	60.56	1E-161	451
SMil_00011064	AT1G05010.1	DOXC	72.14	2.00E-163	451	Os09g27820	DOXC	71.16	3E-152	423
SMil_00012032	AT1G04350.1	DOXC	41.9	3.00E-95	281	Os03g48430	DOXC	41.76	4E-86	258
SMil_00012033	AT1G06620.1	DOXC	46.89	1.00E-120	346	Os03g48430	DOXC	43.73	2E-98	290
SMil_00012259	AT1G17020.1	DOXC	53.07	1.00E-144	407	Os10g40880	DOXC	43.87	3E-100	294
SMil_00013059	AT5G24530.1	DOXC	65.79	3.00E-163	452	Os03g03034	DOXC	60.36	3E-152	424
SMil_00013081	AT1G49390.1	DOXC	51.62	2.00E-115	332	Os06g08032	DOXC	40.91	4E-79	239
SMil_00013222	AT3G13610.1	DOXC	47.14	3.00E-119	342	Os03g03034	DOXC	33.23	3E-55	177
SMil_00013632	AT1G52800.1	DOXC	45.53	4.00E-75	223	Os08g32170	DOXC	29.63	2E-30	107
SMil_00013634	AT1G52820.1	DOXC	42.63	1.00E-56	174	Os05g50090	DOXC	39.42	9E-35	119
SMil_00013635	AT1G52820.1	DOXC	48.73	5.00E-98	288	Os08g32160	DOXC	27.27	5E-40	137
SMil_00013636	AT1G52820.1	DOXC	59.49	6.00E-132	371	Os08g32170	DOXC	32.9	2E-58	183
SMil_00013637	AT1G52820.1	DOXC	50	2.00E-110	315	Os08g32170	DOXC	29.35	1E-44	147
SMil_00013638	AT1G15550.1	DOXC	59.88	8.00E-141	397	Os01g08220	DOXC	41.35	3E-87	261
SMil_00013688	AT5G07480.1	DOXC	45.88	3.00E-52	164	Os04g57160	DOXC	41.62	3E-45	145
SMil_00013745	AT3G19000.1	DOXC	57.46	2.00E-143	404	Os03g42130	DOXC	52.82	9E-133	377
SMil_00013746	AT3G19000.1	DOXC	52.96	2.00E-114	328	Os03g42130	DOXC	52.59	8E-106	306
SMil_00014171	AT4G10490.1	DOXC	33.94	2.00E-30	107	Os04g55070	DOXC	56.76	4E-89	258

SMil_00014304	AT1G78440.1	DOXC	56.57	4.00E-82	240	Os01g55240	DOXC	58.25	9E-81	236
SMil_00014612	AT4G21200.1	DOXC	52.52	4.00E-129	365	Os04g44150	DOXC	52.91	8E-124	353
SMil_00014618	AT3G51240.1	DOXC	70.55	4.00E-160	444	Os04g56700	DOXC	69.72	9E-153	426
SMil_00014774	AT1G14130.1	DOXC	44.52	1.00E-84	250	Os04g39980	DOXC	41.86	2E-81	241
SMil_00015044	AT1G17020.1	DOXC	38.13	7.00E-56	178	Os01g24980	DOXC	41.46	8E-62	192
SMil_00016062	AT1G06620.1	DOXC	45.79	2.00E-104	305	Os03g48430	DOXC	39.4	9E-85	254
SMil_00016109	AT1G06620.1	DOXC	49.44	8.00E-110	319	Os03g48430	DOXC	43.72	3E-90	269
SMil_00016110	AT1G06620.1	DOXC	46.8	8.00E-117	337	Os03g48430	DOXC	43.84	1E-101	298
SMil_00016112	AT5G43440.1	DOXC	58.56	3.00E-34	115	Os04g10350	DOXC	57.66	3E-34	115
SMil_00016116	AT1G06620.1	DOXC	52.02	4.00E-137	389	Os03g48430	DOXC	46.21	7E-107	312
SMil_00016123	AT4G21200.1	DOXC	60.99	5.00E-82	240	Os04g44150	DOXC	51.31	2E-66	201
SMil_00016302	AT5G51810.1	DOXC	48.34	3.00E-85	253	Os01g66100	DOXC	50.76	1E-77	234
SMil_00016603	AT1G14130.1	DOXC	45.78	2.00E-70	213	Os04g39980	DOXC	41.13	1E-63	196
SMil_00016763	AT1G52800.1	DOXC	44.13	2.00E-103	299	Os08g32170	DOXC	32.23	3E-53	170
SMil_00016764	AT1G52800.1	DOXC	55.71	5.00E-129	363	Os08g32160	DOXC	32.9	8E-52	166
SMil_00016766	AT1G52800.1	DOXC	48.11	2.00E-83	244	Os08g32170	DOXC	30.28	3E-37	125
SMil_00016767	AT1G52800.1	DOXC	47.28	2.00E-64	196	Os08g32170	DOXC	29.69	8E-29	103
SMil_00017774	AT1G30040.1	DOXC	60.13	1.00E-138	390	Os01g55240	DOXC	56.62	4E-129	365
SMil_00018080	AT1G04380.1	DOXC	52.91	5.00E-65	196	Os04g10350	DOXC	47.2	2E-52	164
SMil_00018082	AT1G06620.1	DOXC	45.38	8.00E-106	308	Os03g48430	DOXC	41.42	2E-84	254
SMil_00018100	AT1G06620.1	DOXC	47.75	6.00E-120	345	Os03g48430	DOXC	43.47	1E-97	288
SMil_00018122	AT4G10490.1	DOXC	39.76	2.00E-54	172	Os08g37456	DOXC	43.44	1E-67	206
SMil_00018123	AT4G10500.1	DOXC	37.36	2.00E-72	222	Os08g37456	DOXC	42.59	4E-83	249
SMil_00018124	AT2G36690.1	DOXC	40.65	1.00E-65	204	Os08g37456	DOXC	45.1	1E-79	239
SMil_00018452	AT2G36690.1	DOXC	61.93	6.00E-165	459	Os08g44590	DOXC	52.1	2E-132	377
SMil_00018601	AT3G51240.1	DOXC	74.48	0	537	Os04g56700	DOXC	73.42	0	504

SMil_00018668	AT1G04380.1	DOXC	49.72	5.00E-61	191	Os04g10350	DOXC	46.91	3E-56	179
SMil_00018669	AT5G59530.1	DOXC	49.72	4.00E-123	353	Os03g48430	DOXC	45.33	3E-107	313
SMil_00018670	AT5G43440.1	DOXC	47.13	8.00E-54	167	Os04g10350	DOXC	47.97	2E-52	163
SMil_00019079	AT1G30040.1	DOXC	71.54	3.00E-70	211	Os01g55240	DOXC	69.01	2E-67	204
SMil_00019091	AT5G20400.1	DOXC	53.68	1.00E-96	280	Os02g17940	DOXC	48.07	9E-81	239
SMil_00019092	AT5G20400.1	DOXC	45.32	5.00E-76	231	Os02g17940	DOXC	39.93	2E-62	196
SMil_00019093	AT5G20400.1	DOXC	47.64	3.00E-54	169	Os02g17940	DOXC	39.06	7E-39	129
SMil_00019233	AT4G10490.1	DOXC	39.57	6.00E-63	196	Os08g37456	DOXC	44.07	4E-89	263
SMil_00019234	AT4G10500.1	DOXC	37.94	4.00E-64	201	Os08g37456	DOXC	43.59	2E-90	268
SMil_00019235	AT1G78550.1	DOXC	44.44	4.00E-46	148	Os03g03034	DOXC	44	4E-50	158
SMil_00019259	AT1G52820.1	DOXC	52.66	2.00E-120	342	Os08g32170	DOXC	33.33	1E-60	189
SMil_00019454	AT1G14130.1	DOXC	47.22	3.00E-20	76.6	Os04g39980	DOXC	48.57	3E-22	82
SMil_00019542	AT1G52800.1	DOXC	46.41	6.00E-51	163	Os05g50090	DOXC	37.5	2E-27	102
SMil_00019654	AT1G52800.1	DOXC	48.65	5.00E-66	201	Os08g32170	DOXC	30.94	4E-29	105
SMil_00019970	AT1G52790.1	DOXC	38.19	6.00E-78	233	Os08g32170	DOXC	29.9	1E-47	155
SMil_00020342	AT2G44800.1	DOXC	35.56	5.00E-64	202	Os08g37456	DOXC	42.3	5E-87	261
SMil_00020663	AT1G30040.1	DOXC	46.41	8.00E-91	266	Os01g55240	DOXC	44.44	8E-85	250
SMil_00020998	AT1G14130.1	DOXC	44.86	5.00E-91	266	Os04g39980	DOXC	44.09	1E-83	247
SMil_00021184	AT4G10490.1	DOXC	35.49	4.00E-52	169	Os08g37456	DOXC	36.22	9E-62	193
SMil_00021185	AT2G36690.1	DOXC	35.88	5.00E-75	229	Os08g37456	DOXC	42.9	2E-92	273
SMil_00021186	AT4G10490.1	DOXC	36.15	1.00E-49	160	Os08g37456	DOXC	38	4E-58	181
SMil_00021188	AT4G10490.1	DOXC	42.63	5.00E-46	148	Os08g37456	DOXC	42.22	4E-47	150
SMil_00021251	AT1G04350.1	DOXC	45.45	2.00E-54	169	Os08g30080	DOXC	44.62	2E-47	152
SMil_00021366	AT1G30040.1	DOXC	56.05	3.00E-93	271	Os01g55240	DOXC	52.68	8E-78	231
SMil_00022077	AT5G05600.1	DOXC	58.61	7.00E-125	355	Os05g03640	DOXC	54.93	7E-115	329
SMil_00022092	AT1G77330.1	DOXC	67.42	3.00E-146	407	Os05g05680	DOXC	62.82	5E-126	355

SMil_00022299	AT1G30040.1	DOXC	61.11	4.00E-121	343	Os01g55240	DOXC	60.75	9E-112	319
SMil_00022446	AT2G36690.1	DOXC	34.3	1.00E-49	163	Os08g37456	DOXC	38.67	2E-68	211
SMil_00022937	AT1G35190.1	DOXC	64.29	3.00E-97	279	Os01g09300	DOXC	56.61	6E-104	297
SMil_00022938	AT1G35190.1	DOXC	57.02	7.00E-119	340	Os01g09300	DOXC	54.76	1E-125	359
SMil_00023724	AT1G14130.1	DOXC	53.77	7.00E-108	309	Os04g39980	DOXC	48.81	2E-95	277
SMil_00023785	AT1G17020.1	DOXC	51.59	6.00E-118	339	Os10g41020	DOXC	40	8E-86	257
SMil_00024026	AT2G30830.1	DOXC	38.1	1.00E-57	182	Os03g48430	DOXC	37.57	9E-49	159
SMil_00024092	AT4G10490.1	DOXC	35.69	2.00E-66	206	Os07g07410	DOXC	33.24	1E-66	207
SMil_00024282	AT1G49390.1	DOXC	48.96	2.00E-121	347	Os06g08023	DOXC	41.3	2E-92	274
SMil_00024284	AT5G20400.1	DOXC	52.05	2.00E-119	343	Os02g17940	DOXC	46.06	3E-98	288
SMil_00024563	AT4G21200.1	DOXC	56.85	3.00E-140	395	Os04g44150	DOXC	50.29	1E-116	335
SMil_00024988	AT5G58660.1	DOXC	47.78	7.00E-98	286	Os04g33360	DOXC	39.62	2E-64	201
SMil_00025213	AT2G44800.1	DOXC	35.49	3.00E-65	203	Os08g37456	DOXC	42.6	3E-87	259
SMil_00025718	AT3G51240.1	DOXC	75.96	0	546	Os04g56700	DOXC	73.85	0	513
SMil_00025860	AT1G77330.1	DOXC	69.21	2.00E-161	445	Os05g05680	DOXC	59.94	3E-134	376
SMil_00026100	AT5G07200.1	DOXC	39.43	2.00E-49	160	Os03g42130	DOXC	36.99	5E-43	142
SMil_00026530	AT5G51810.1	DOXC	59.88	1.00E-157	440	Os03g63970	DOXC	55.56	1E-136	387
SMil_00026896	AT1G77330.1	DOXC	67.74	2.00E-145	405	Os05g05680	DOXC	61.66	2E-123	348
SMil_00027265	AT5G24530.1	DOXC	63.85	2.00E-150	418	Os03g03034	DOXC	63.27	2E-149	416
SMil_00027610	AT5G24530.1	DOXC	39.62	2.00E-58	183	Os03g03034	DOXC	41.04	3E-59	184
SMil_00027988	AT5G24530.1	DOXC	39.76	2.00E-71	219	Os10g39140	DOXC	38.08	5E-70	215
SMil_00028133	AT4G10490.1	DOXC	33.04	2.00E-29	105	Os04g55070	DOXC	56.77	1E-93	270
SMil_00028460	AT1G47990.1	DOXC	49.52	2.00E-101	294	Os01g55240	DOXC	43.71	1E-76	230
SMil_00028466	AT1G52820.1	DOXC	62.12	7.00E-87	252	Os08g32170	DOXC	36.68	8E-43	139
SMil_00029330	AT1G04380.1	DOXC	54.94	3.00E-63	191	Os04g10350	DOXC	52.6	1E-59	183
SMil_00029331	AT1G06620.1	DOXC	46.41	5.00E-115	333	Os03g48430	DOXC	41.69	8E-96	284

SMil_00029346	AT1G52820.1	DOXC	58.23	6.00E-136	381	Os08g32170	DOXC	32.58	4E-61	190
SMil_00029434	AT4G22880.1	DOXC	86.93	2.00E-97	279	Os01g27490	DOXC	57.42	1E-66	201
SMil_00029593	AT3G21420.1	DOXC	73.58	6.00E-107	305	Os01g70930	DOXC	65.97	3E-93	270
SMil_00030201	AT4G25420.1	DOXC	67.14	7.00E-179	497	Os03g63970	DOXC	59.25	4E-160	449
SMil_00030383	AT3G60290.1	DOXC	41.83	4.00E-63	197	Os04g49210	DOXC	39.31	3E-62	194
SMil_00000263	AT1G22950.1	unclassified	63.58	9.00E-164	459	Os01g31770	unclassified	62.63	4E-177	493
SMil_00006313	AT1G68080.1	unclassified	49.75	2.00E-112	328	Os03g13550	unclassified	42.81	7E-76	231
SMil_00011070	AT3G63290.1	unclassified	25.32	2.00E-16	73.2	Os05g48230	unclassified	62.17	2E-164	461

Supplementary Table 4. The gene expression of 2OGDs in different organs, tissues, and following MeJA treatment.

Gene_ID	Clades	Classes	Periderm	Phloem	xylem	Flower	Stem	Leaf	Root	MeJA-0	MeJA-12
SMil_00011064	ACO	DOXC	26.63	55.38	30.69	2082.68	303.86	316.91	108.12	1159.81	2034.96
SMil_00022077	ACO	DOXC	10.04	4.58	1.92	20.42	51.19	1.09	44.51	3.00	182.02
SMil_00005833	ACO	DOXC	7.33	2.32	0.99	5.36	12.01	13.68	22.69	60.67	207.65
SMil_00006040	ACO	DOXC	10.95	6.98	7.69	58.06	25.26	8.19	11.20	9.34	13.41
SMil_00026896	ACO	DOXC	0.12	0.00	0.09	0.22	2.71	0.00	3.20	0.11	0.48
SMil_00022092	ACO	DOXC	0.76	0.42	0.30	0.20	1.14	0.25	1.69	0.35	0.55
SMil_00007557	ACO	DOXC	0.24	3.41	2.40	0.14	3.35	0.00	1.67	0.03	0.00
SMil_00025860	ACO	DOXC	2.83	1.33	0.26	0.00	0.03	0.00	0.34	0.04	0.00
SMil_00016766	AOP	DOXC	49.61	52.69	7.71	0.05	3.55	0.00	172.47	0.00	0.00
SMil_00019970	AOP	DOXC	34.78	18.96	24.66	0.17	0.22	0.18	63.96	0.15	0.20
SMil_00019654	AOP	DOXC	27.33	27.10	25.69	66.41	29.28	8.72	29.37	11.50	15.14
SMil_00013632	AOP	DOXC	9.59	8.36	0.47	0.04	0.82	0.00	11.16	0.00	0.00
SMil_00019259	AOP	DOXC	6.54	0.75	0.08	0.06	0.37	0.00	10.35	0.00	0.00
SMil_00029346	AOP	DOXC	15.45	17.89	14.95	0.02	3.80	0.63	8.41	0.66	0.68
SMil_00013636	AOP	DOXC	10.94	15.86	12.65	0.04	0.46	0.40	4.56	0.27	0.48
SMil_00028466	AOP	DOXC	0.59	0.72	0.57	0.00	0.90	0.00	3.83	0.00	0.02
SMil_00016763	AOP	DOXC	2.94	3.00	0.14	0.00	0.12	0.00	2.41	0.00	0.00
SMil_00013637	AOP	DOXC	1.36	0.20	0.00	3.68	3.74	1.89	1.62	2.04	3.12
SMil_00019542	AOP	DOXC	0.37	0.58	0.29	2.10	0.99	0.00	0.68	0.25	0.42
SMil_00000574	AOP	DOXC	13.17	4.86	2.38	1.35	1.69	11.17	0.53	5.66	0.07
SMil_00016767	AOP	DOXC	0.00	0.00	0.03	0.21	0.07	0.00	0.06	0.00	0.00
SMil_00013634	AOP	DOXC	0.38	0.06	0.05	0.00	0.10	0.00	0.00	0.00	0.00
SMil_00013635	AOP	DOXC	0.43	0.22	0.31	0.00	0.01	0.00	0.00	0.00	0.00

SMil_00016764	AOP	DOXC	0.00	0.00	0.00	0.00	0.00	0.00	0.00	0.00	0.00
SMil_00012259	CODM/NCS	DOXC	412.59	143.92	129.72	265.95	1.06	0.48	138.44	1.44	0.54
SMil_00024284	CODM/NCS	DOXC	30.30	20.98	18.39	70.31	22.97	12.27	14.92	9.81	10.18
SMil_00019091	CODM/NCS	DOXC	19.05	17.08	13.94	6.99	5.21	4.82	10.14	2.27	2.41
SMil_00013081	CODM/NCS	DOXC	124.36	36.50	24.41	37.57	6.93	2.40	7.29	4.38	29.65
SMil_00024282	CODM/NCS	DOXC	135.18	55.22	19.65	3.55	4.23	0.39	2.55	0.29	5.19
SMil_00019092	CODM/NCS	DOXC	1.55	4.33	0.25	9.57	0.35	0.00	0.19	0.00	0.00
SMil_00008970	CODM/NCS	DOXC	0.26	2.82	0.76	8.59	3.87	1.35	0.15	0.69	0.21
SMil_00019093	CODM/NCS	DOXC	1.13	0.62	0.08	1.02	0.00	0.00	0.14	0.00	0.00
SMil_00015044	CODM/NCS	DOXC	1.91	0.00	0.00	0.00	0.01	0.00	0.09	0.00	0.00
SMil_00029593	CODM/NCS	DOXC	0.10	1.19	0.41	0.78	0.85	0.00	0.08	0.06	0.16
SMil_00023785	CODM/NCS	DOXC	0.80	0.05	0.00	2.52	0.79	3.89	0.06	8.59	23.04
SMil_00012032	D4H/GSLOH/BX6	DOXC	45.90	24.78	8.29	5.37	114.20	0.81	270.35	5.60	79.07
SMil_00018668	D4H/GSLOH/BX6	DOXC	73.56	23.72	0.39	22.87	33.40	2.62	52.94	0.00	0.03
SMil_00018669	D4H/GSLOH/BX6	DOXC	27.87	16.58	16.02	22.87	33.40	2.62	52.94	11.53	7.06
SMil_00029331	D4H/GSLOH/BX6	DOXC	41.15	9.12	3.67	4.51	14.41	3.65	13.68	3.41	26.07
SMil_00018670	D4H/GSLOH/BX6	DOXC	8.61	4.37	3.10	9.53	38.55	40.09	12.84	24.42	42.15
SMil_00016110	D4H/GSLOH/BX6	DOXC	17.93	5.92	3.64	25.01	79.04	35.64	11.98	60.41	482.59
SMil_00029330	D4H/GSLOH/BX6	DOXC	16.04	1.74	0.13	4.13	13.12	4.81	3.80	8.93	25.21
SMil_00016116	D4H/GSLOH/BX6	DOXC	2.06	2.25	1.87	8.11	11.08	2.44	2.14	9.56	5.29
SMil_00024026	D4H/GSLOH/BX6	DOXC	9.99	6.99	5.76	3.26	1.19	0.24	1.76	0.59	8.18
SMil_00018100	D4H/GSLOH/BX6	DOXC	50.15	9.09	0.66	0.02	0.02	0.00	1.23	0.00	0.00
SMil_00018082	D4H/GSLOH/BX6	DOXC	0.21	0.03	0.00	0.00	0.04	0.00	0.50	0.00	0.00
SMil_00018080	D4H/GSLOH/BX6	DOXC	0.42	0.14	0.00	0.00	0.00	0.00	0.16	0.00	0.00
SMil_00016109	D4H/GSLOH/BX6	DOXC	0.27	0.00	0.00	0.14	0.81	0.26	0.11	0.26	0.79
SMil_00016062	D4H/GSLOH/BX6	DOXC	1.11	0.21	0.05	0.06	31.11	0.08	0.02	0.03	0.02

SMil_00021251	D4H/GSLOH/BX6	DOXC	0.53	0.13	0.00	0.28	0.20	0.00	0.00	0.41	0.72
SMil_00012033	D4H/GSLOH/BX6	DOXC	0.00	0.00	0.00	0.00	0.00	0.00	0.00	0.00	0.00
SMil_00016112	D4H/GSLOH/BX6	DOXC	0.00	0.00	0.00	0.00	0.00	0.00	0.00	0.00	0.00
SMil_00023724	DAO	DOXC	14.98	17.16	13.44	9.22	14.15	31.41	16.59	16.74	12.55
SMil_00014774	DAO	DOXC	0.80	0.12	0.20	0.33	0.13	0.31	0.16	0.18	0.14
SMil_00016603	DAO	DOXC	1.08	0.13	0.15	0.14	0.01	0.00	0.01	0.00	0.00
SMil_00020998	DAO	DOXC	3.57	0.81	0.19	0.10	0.02	0.00	0.00	0.03	0.05
SMil_00019454	DAO	DOXC	0.50	0.07	0.00	0.00	0.00	0.00	0.00	0.00	0.00
SMil_00013746	F3H	DOXC	16.95	5.65	9.87	74.06	65.58	156.30	73.71	314.14	43.32
SMil_00022937	F3H	DOXC	69.50	58.53	58.63	31.66	43.89	78.95	60.90	9.78	9.35
SMil_00005410	F3H	DOXC	12.97	16.55	14.78	18.09	15.72	12.51	12.88	6.15	23.75
SMil_00008540	F3H	DOXC	22.65	18.19	16.25	10.55	6.50	2.62	8.02	9.50	3.40
SMil_00013745	F3H	DOXC	39.67	3.56	1.27	27.81	21.64	8.04	5.66	11.78	3.34
SMil_00028133	F3H	DOXC	4.37	2.92	3.25	0.31	3.46	0.00	1.99	0.26	1.06
SMil_00025718	F3H	DOXC	2.72	0.15	0.06	93.37	21.03	28.03	1.07	95.73	7.44
SMil_00014171	F3H	DOXC	7.07	1.30	1.38	0.39	0.59	0.00	0.57	0.05	0.35
SMil_00014618	F3H	DOXC	0.00	0.00	0.00	20.91	0.03	0.00	0.20	0.04	0.00
SMil_00008541	F3H	DOXC	0.00	0.00	0.00	0.00	0.00	0.00	0.00	0.00	0.00
SMil_00018601	F3H	DOXC	0.00	0.00	0.00	3.38	0.00	0.00	0.00	0.00	0.02
SMil_00022938	F3H	DOXC	0.00	0.00	0.00	0.00	0.00	0.00	0.00	15.80	18.22
SMil_00029434	FLS/ANS	DOXC	0.00	0.00	0.00	570.96	0.50	0.00	2.08	0.33	0.00
SMil_00003616	FLS/ANS	DOXC	0.00	0.00	0.29	70.06	65.44	268.86	0.66	241.61	14.31
SMil_00004980	FLS/ANS	DOXC	0.24	0.00	0.04	2.91	0.30	0.32	0.20	2.54	0.83
SMil_00026530	GA20ox	DOXC	0.04	1.95	0.13	1.27	24.80	0.00	19.99	0.00	0.00
SMil_00008090	GA20ox	DOXC	1.50	1.94	2.27	45.86	6.58	2.03	2.28	0.67	0.38
SMil_00026100	GA20ox	DOXC	0.95	0.12	0.09	0.06	1.65	0.00	1.57	0.00	0.02

SMil_00030201	GA20ox	DOXC	3.34	1.96	2.37	2.63	2.46	0.44	0.67	2.21	3.44
SMil_00016302	GA20ox	DOXC	0.24	0.03	0.00	2.19	0.57	0.46	0.22	5.71	0.33
SMil_00010684	GA20ox	DOXC	0.23	0.04	0.10	0.15	0.33	0.00	0.00	0.00	0.00
SMil_00017774	GA2ox	DOXC	8.37	14.85	15.37	25.92	12.49	5.47	39.93	8.09	0.51
SMil_00022299	GA2ox	DOXC	10.03	11.15	13.89	10.67	7.92	6.82	28.91	5.38	0.61
SMil_00024988	GA2ox	DOXC	2.03	1.85	4.21	106.42	12.53	11.07	19.15	17.67	30.98
SMil_00004141	GA2ox	DOXC	2.78	3.16	2.81	3.08	8.13	0.28	14.54	3.93	1.24
SMil_00005648	GA2ox	DOXC	0.02	0.12	0.02	190.12	5.64	1.58	8.20	0.00	0.09
SMil_00024563	GA2ox	DOXC	0.09	0.54	0.34	1.46	7.77	1.29	1.62	3.46	0.33
SMil_00028460	GA2ox	DOXC	12.76	2.89	0.74	0.00	0.05	0.31	0.87	0.25	0.06
SMil_00016123	GA2ox	DOXC	0.36	0.00	0.13	0.26	0.42	0.00	0.48	0.12	0.00
SMil_00014304	GA2ox	DOXC	0.00	0.00	0.06	0.03	0.04	1.38	0.23	2.85	0.30
SMil_00020663	GA2ox	DOXC	0.00	0.00	0.00	0.00	0.03	0.54	0.19	3.11	0.30
SMil_00021366	GA2ox	DOXC	0.14	0.00	0.04	4.00	12.25	6.89	0.07	30.77	13.05
SMil_00014612	GA2ox	DOXC	0.00	0.00	0.00	0.04	0.00	0.00	0.00	0.00	0.00
SMil_00019079	GA2ox	DOXC	0.00	0.00	0.00	0.00	0.00	0.00	0.00	0.16	0.13
SMil_00013638	GA3ox	DOXC	0.00	0.00	0.25	6.88	6.72	0.00	1.75	0.76	0.13
SMil_00003886	GA3ox	DOXC	5.83	2.94	3.42	1.16	0.00	0.00	0.41	0.20	0.13
SMil_00024092	H6H	DOXC	29.73	17.98	25.56	7.32	0.00	0.17	7.46	0.10	0.29
SMil_00027988	H6H	DOXC	3.22	3.44	0.70	0.31	0.13	0.00	0.50	0.03	0.39
SMil_00027610	H6H	DOXC	6.52	7.84	1.75	0.00	0.00	0.00	0.45	0.00	0.04
SMil_00001764	P4H	DOXB	77.07	58.74	53.39	41.30	63.39	58.63	40.01	59.01	51.78
SMil_00013421		DOXA	2.41	1.82	1.53	7.52	3.86	0.33	31.46	1.01	1.58
SMil_00001015	P4H	DOXB	84.84	46.27	36.29	27.27	43.79	64.03	29.74	21.18	25.44
SMil_00004726	P4H	DOXB	32.33	28.55	25.51	50.98	43.14	16.69	23.68	27.92	39.97
SMil_00018996	P4H	DOXB	27.22	19.76	19.76	16.51	17.73	32.92	19.14	14.63	15.65

SMil_00021498		DOXA	3.25	8.85	7.12	47.49	28.31	36.81	18.91	45.47	11.42
SMil_00000263		Unclassified	17.64	15.34	14.80	16.52	14.81	10.38	12.30	8.61	10.64
SMil_00021036	P4H	DOXB	30.83	26.30	33.99	13.84	18.96	22.58	10.80	6.15	4.54
SMil_00006860	P4H	DOXB	12.12	13.70	10.85	7.94	8.01	4.30	8.12	2.14	5.12
SMil_00001058		DOXA	7.31	6.11	6.37	4.90	4.41	1.82	6.89	1.91	2.04
SMil_00011070		Unclassified	10.03	7.86	7.33	5.90	2.13	3.60	3.72	1.29	2.67
SMil_00006313		Unclassified	3.58	3.38	4.45	4.38	5.83	7.24	2.92	2.89	3.87
SMil_00013255	P4H	DOXB	5.79	4.38	4.68	3.53	3.07	2.68	2.47	1.32	1.28
SMil_00023889	P4H	DOXB	0.00	0.00	0.01	0.01	0.53	0.25	0.13	0.05	0.00
SMil_00013059	S3H	DOXC	1.73	5.07	0.57	6.07	31.46	0.00	20.04	0.56	0.17
SMil_00027265	S3H	DOXC	11.65	13.36	7.14	1.96	2.62	2.18	2.33	1.05	4.12
SMil_00020342	Unknown	DOXC	109.93	69.27	19.83	15.58	297.68	0.96	146.22	9.13	21.44
SMil_00021188	Unknown	DOXC	0.15	0.13	14.54	55.69	109.70	18.64	40.98	23.90	4.41
SMil_00009012	Unknown	DOXC	3.18	0.60	0.50	16.18	16.59	5.36	39.26	0.03	0.31
SMil_00025213	Unknown	DOXC	0.45	0.12	0.06	0.17	14.16	0.00	29.19	0.03	3.92
SMil_00021185	Unknown	DOXC	24.86	24.24	21.97	72.98	47.18	6.79	23.53	29.89	13.76
SMil_00018122	Unknown	DOXC	0.06	0.04	0.50	49.92	37.22	19.89	8.72	34.06	4.17
SMil_00013688	Unknown	DOXC	21.13	17.71	19.66	14.21	9.32	12.23	8.31	5.28	7.46
SMil_00021184	Unknown	DOXC	0.82	0.50	1.53	7.99	16.69	1.37	4.95	2.80	2.08
SMil_00019234	Unknown	DOXC	0.29	0.18	0.03	0.00	1.49	0.00	3.40	0.00	0.10
SMil_00019233	Unknown	DOXC	0.20	0.15	0.12	0.02	0.07	0.00	2.31	0.02	0.02
SMil_00018452	Unknown	DOXC	7.28	8.47	5.57	0.70	1.24	0.36	1.67	2.76	0.90
SMil_00018124	Unknown	DOXC	9.41	4.10	3.01	28.31	0.04	0.00	0.23	0.00	31.42
SMil_00022446	Unknown	DOXC	0.00	0.02	0.00	0.00	0.11	0.00	0.18	0.00	0.00
SMil_00018123	Unknown	DOXC	17.80	3.00	0.00	0.08	0.02	0.00	0.12	0.00	0.00
SMil_00019235	Unknown	DOXC	0.00	0.00	0.00	0.00	0.05	0.00	0.10	0.00	0.00

SMil_00030383	Unknown	DOXC	0.09	0.10	0.13	1.80	1.73	9.52	0.03	3.99	1.51
SMil_00007843	Unknown	DOXC	0.02	0.00	0.00	0.00	0.00	0.00	0.00	0.15	0.00
SMil_00009013	Unknown	DOXC	0.00	0.00	0.00	0.00	0.00	0.00	0.00	0.43	0.30
SMil_00013222	Unknown	DOXC	0.00	0.00	0.00	0.04	0.00	0.15	0.00	0.18	0.57

Supplementary Table5. Candidate 2OGDs related to tanshinone biosynthesis in *S. miltiorrhiza*.

**Gene_IDs**	**Gene_name**	**Clades**	**Classes**	**Periderm**	**Phloem**	**xylem**	**Flower**	**Stem**	**Leaf**	**Root**	**MeJA-0**	**MeJA-12**
SMil_00024282	2OGD-1	CODM/NCS	DOXC	135.18	55.22	19.65	3.55	4.23	0.39	2.55	0.29	5.19
SMil_00020342	2OGD-2	Unknown	DOXC	109.93	69.27	19.83	15.58	297.68	0.96	146.22	9.13	21.44
SMil_00018668	2OGD-3	D4H/GSLOH/BX6	DOXC	73.56	23.72	0.39	22.87	33.40	2.62	52.94	0.00	0.03
SMil_00018100	2OGD-4	D4H/GSLOH/BX6	DOXC	50.15	9.09	0.66	0.02	0.02	0.00	1.23	0.00	0.00
SMil_00012032	2OGD-5	D4H/GSLOH/BX6	DOXC	45.90	24.78	8.29	5.37	114.20	0.81	270.35	5.60	79.07
SMil_00029331	2OGD-6	D4H/GSLOH/BX6	DOXC	41.15	9.12	3.67	4.51	14.41	3.65	13.68	3.41	26.07
SMil_00013745	2OGD-7	F3H	DOXC	39.67	3.56	1.27	27.81	21.64	8.04	5.66	11.78	3.34
SMil_00016110	2OGD-8	D4H/GSLOH/BX6	DOXC	17.93	5.92	3.64	25.01	79.04	35.64	11.98	60.41	482.59
SMil_00018123	2OGD-9	Unknown	DOXC	17.80	3.00	0.00	0.08	0.02	0.00	0.12	0.00	0.00
SMil_00029330	2OGD-10	D4H/GSLOH/BX6	DOXC	16.04	1.74	0.13	4.13	13.12	4.81	3.80	8.93	25.21
SMil_00000574	2OGD-11	AOP	DOXC	13.17	4.86	2.38	1.35	1.69	11.17	0.53	5.66	0.07
SMil_00028460	2OGD-12	GA2ox	DOXC	12.76	2.89	0.74	0.00	0.05	0.31	0.87	0.25	0.06
SMil_00022077	2OGD-13	ACO	DOXC	10.04	4.58	1.92	20.42	51.19	1.09	44.51	3.00	182.02

Supplementary Table6. Different tanshinone metabolites analysed from 2OGD5-silencing lines and control lines.

Compoud name	RT(min)	M+H	MS/MS	Molecular formula	Molecular weight	CAS	PK1	PK2	PK3	Average PK	Standard PK	2OGD-5	Difference ratio (2OGD5/
Salvisyrianone	9.07	313	313,295,277, 267,253,241, 227	C20H24O3	312	**250691-57-7**	6.77E+06	5.71E+06	7.69E+06	6.72E+06	9.91E+05	9.83E+06	1.46
Cryptojaponol	7.24	331	331,313,303, 301,295,285, 283	C21H30O3	330	-	1.05E+06	8.65E+05	1.06E+06	9.92E+05	1.10E+05	1.24E+06	1.25
Trijuganone B	8.38	281	281,263,248, 235,220,192	C18H16O3	280	**126979-84-8**	2.65E+05	2.38E+05	5.70E+05	3.58E+05	1.84E+05	3.91E+05	1.09

Danshenxinkun A	6.71	297	297,279,261, 251,233,205, 190	C18H16O4	296	**65907-75-7**	2.17E+06	3.12E+06	2.59E+06	2.63E+06	4.76E+05	2.86E+06	1.09
3α-Hydroxymethylenetanshinquinone	5.66	295	295,277,267, 251,249,221	C18H14O4	294	**83145-47-5**	9.00E+05	7.09E+05	6.14E+05	7.41E+05	1.46E+05	7.79E+05	1.05
1, 2-Didehydrotanshinone V	6.13	313	313,295,280, 277,267,253, 249,225	C19H20O4	312	-	9.94E+05	9.35E+05	7.45E+05	8.91E+05	1.30E+05	8.41E+05	0.94

Tanshinol B	7.4	297	297,279,261, 233,218,205, 190	C18H16O4	296	**189290-30-0**	1.33E+07	1.04E+07	1.00E+07	1.12E+07	1.80E+06	1.02E+07	0.91
Hydroxytanshinone IIA	6.4	311	311,293,278, 247,219,204, 191	C19H18O4	310	**18887-18-8**	2.76E+05	4.74E+05	3.24E+05	3.58E+05	1.03E+05	3.13E+05	0.87
Methylenetanshinquinone	7.4	279	279,261,233, 205,201,190, 149	C18H14O3	278	**67656-29-5**	2.18E+05	2.87E+05	2.83E+05	2.63E+05	3.87E+04	2.24E+05	0.85

Tanshinone IIB	7.15	311	311,293,278,<br>275,265,251,<br>247	$C_{19}H_{18}O_4$	310	**17397-93-2**	2.56E+06	3.45E+06	2.76E+06	2.92E+06	4.67E+05	2.35E+06	0.80
trijuganone C	7.53	341	341,281,263,<br>235	$C_{20}H_{20}O_5$	340	**135247-94-8**	3.17E+06	2.54E+06	2.86E+06	2.86E+06	3.15E+05	2.29E+06	0.80
Tanshinone I	8.91	277	277,259,235,<br>249,221,193,<br>178	$C_{18}H_{12}O_3$	276	**568-73-0**	1.11E+07	1.42E+07	1.09E+07	1.21E+07	1.85E+06	9.64E+06	0.80

1, 2-Didehydrotanshinone IIA	9.63	293	279,261,233, 218,205,190, 169	$C_{19}H_{16}O_3$	292	**119963-50-7**	1.62E+06	2.12E+06	1.66E+06	1.80E+06	2.78E+05	1.33E+06	0.74
15, 16-Dihydrotanshinone I	8.08	279	279,261,233, 218,205,190, 169	C18H14O3	278	**87205-99-0**	1.16E+07	1.20E+07	1.20E+07	1.19E+07	2.31E+05	8.62E+06	0.73
Methyltanshinonate	8.42	339	339,279,261, 233,205,190, 149	C20H18O5	338	**135355-72-5**	6.70E+06	7.88E+06	7.17E+06	7.25E+06	5.94E+05	4.77E+06	0.66

1R-hydroxymiltirone	7.77	299	299,281,266, 263,253,221, 193,178	C19H22O3	298	-	2.44E+05	1.54E+05	1.65E+05	1.88E+05	4.91E+04	1.06E+05	0.56
Cryptotanshinone	9.01	297	297,282,279, 267,254,221, 193	C19H20O3	296	**35825-57-1**	2.01E+07	1.38E+07	1.58E+07	1.66E+07	3.22E+06	9.26E+06	0.56
Tanshinone IIA	9.89	295	295,277,267, 253,249,234, 221,206	C19H18O3	294	**568-72-9**	2.71E+06	2.99E+06	2.71E+06	2.80E+06	1.62E+05	1.56E+06	0.56

Sibiriquinone A	7.1	281	281,263,253, 235,207,179	C19H20O2	280	**723300-08-1**	6.91E+04	6.78E+04	5.71E+04	6.47E+04	6.59E+03	3.51E+04	0.54
Tanshinone VI	4.7	297	297,279,261, 251,233,205, 190	C18H16O4	296	**121064-74-2**	5.80E+05	6.06E+05	5.29E+05	5.72E+05	3.92E+04	3.08E+05	0.54
1-Ketoisocryptotanshinone	7.99	311	311,293,283, 275,265,255, 227,221	C19H18O4	310	-	8.98E+04	8.07E+04	5.02E+04	7.36E+04	2.07E+04	3.18E+04	0.43

Tanshindiol B	7.04	313	313,295,277,<br>266,249,235	C18H16O5	312	**97465-70-8**	1.04E+06	7.53E+05	6.23E+05	8.05E+05	2.13E+05	3.36E+05	0.42
Tanshindiol C	7.14	313	313,295,277,<br>266,249,235	C18H16O5	312	**97465-71-9**	4.91E+05	5.32E+05	4.33E+05	4.85E+05	4.97E+04	1.83E+05	0.38
Miltirone	10.09	283	283,265,240,<br>223,208,195,<br>153	C19H22O2	282	**27210-57-7**	3.19E+06	1.28E+06	1.80E+06	2.09E+06	9.87E+05	3.39E+05	0.16
